# Loss of GDE2 leads to complex behavioral changes including memory impairment

**DOI:** 10.1186/s12993-024-00234-1

**Published:** 2024-04-04

**Authors:** Daniel Daudelin, Anna Westerhaus, Nan Zhang, Erica Leyder, Alena Savonenko, Shanthini Sockanathan

**Affiliations:** 1 The Solomon Snyder Department of Neuroscience, The Johns Hopkins School of Medicine, PCTB 1004, 725 N. Wolfe Street, Baltimore, MD 21205 USA; 2 Department of Pathology, The Johns Hopkins University School of Medicine, 558 Ross Research Building, 720 Rutland Avenue, Baltimore, MD 21205 USA; 3 Molecular Microbiology and Immunology Graduate Program in Life Sciences, University of Maryland School of Medicine, 655 W. Baltimore St., Baltimore, MD 21201 USA; 4 Sensory-Motor Neuroscience (SMN), Center for Scientific Review, ICN Review Branch, National Institutes of Health, 6701 Rockledge Drive, Suite 1010-F, Bethesda, MD 20892 USA

**Keywords:** GDE2, Neurodegeneration, Disease, Behavior, Memory impairment, Hyperactivity

## Abstract

**Background:**

Alzheimer’s disease (AD) and amyotrophic lateral sclerosis/frontotemporal dementia (ALS/FTD) are debilitating neurodegenerative diseases for which there are currently no cures. Familial cases with known genetic causes make up less than 10% of these diseases, and little is known about the underlying mechanisms that contribute to sporadic disease. Accordingly, it is important to expand investigations into possible pathways that may contribute to disease pathophysiology. Glycerophosphodiester phosphodiesterase 2 (GDE2 or GDPD5) is a membrane-bound enzyme that acts at the cell surface to cleave the glycosylphosphatidylinositol (GPI)-anchor that tethers distinct proteins to the membrane. GDE2 abnormally accumulates in intracellular compartments in the brain of patients with AD, ALS, and ALS/FTD, indicative of GDE2 dysfunction. Mice lacking GDE2 (*Gde2*KO) show neurodegenerative changes such as neuronal loss, reduced synaptic proteins and synapse loss, and increased Aβ deposition, raising the possibility that GDE2 disruption in disease might contribute to disease pathophysiology. However, the effect of GDE2 loss on behavioral function and learning/memory has not been characterized.

**Results:**

Here, we show that GDE2 is expressed throughout the adult mouse brain in areas including the cortex, hippocampus, habenula, thalamus, and amygdala. *Gde2*KO and WT mice were tested in a set of behavioral tasks between 7 and 16 months of age. Compared to WT, *Gde2*KO mice display moderate hyperactivity that becomes more pronounced with age across a variety of behavioral tests assessing novelty-induced exploratory activity. Additionally, *Gde2*KO mice show reduced startle response, with females showing additional defects in prepulse inhibition. No changes in anxiety-associated behaviors were found, but *Gde2*KOs show reduced sociability. Notably, aged *Gde2*KO mice demonstrate impaired short/long-term spatial memory and cued fear memory/secondary contextual fear acquisition.

**Conclusions:**

Taken together, these observations suggest that loss of GDE2 leads to behavioral deficits, some of which are seen in neurodegenerative disease models, implying that loss of GDE2 may be an important contributor to phenotypes associated with neurodegeneration.

**Supplementary Information:**

The online version contains supplementary material available at 10.1186/s12993-024-00234-1.

## Background

Neurodegenerative diseases such as Alzheimer’s disease (AD) and Amyotrophic Lateral Sclerosis/Frontotemporal Dementia (ALS/FTD) are defined by progressive neurodegeneration and neuronal loss that functionally alters behavior and cognition [1–3]. Studies of familial cases of AD and ALS/FTD have identified genetic causes of disease, such as mutations in *PSEN1 (Presenilin1), APP (Amyloid precursor protein), SOD1 (Superoxide dismutase1)*, and *MAPT (Microtubule associated protein tau)* [1–7]*.* These genetic mutations have been used to generate mouse models that recapitulate many of the cellular and behavioral phenotypes seen in human disease [8, 9]. For example, in the humanized *APP/PS1* double transgenic mouse model of AD that contains disease-associated mutations in the genes encoding *APP* and *PSEN1*, mice exhibit progressive Aβ plaque pathology accompanied by behavioral changes, including hyperactivity, deficits in sensorimotor gating, and the impairment of different types of memory [10–13]. These transgenic mouse models have proved useful in investigating the underlying mechanisms that drive disease pathology in the context of familial AD. However, most cases of AD and ALS/FTD are sporadic, and the underlying genes and proteins that contribute to pathophysiology in these cases are not fully understood [1–3]. Thus, it is important to investigate other potential pathways that may factor in disease pathogenesis.

Glycerophosphodiester phosphodiesterase 2 (GDE2 or GDPD5) is a vertebrate-specific six-transmembrane GPI-anchor cleaving enzyme that cleaves GPI-anchored substrates expressed on the plasma membrane [14–16]. GDE2 is expressed during nervous system development and is required for the differentiation of subsets of cortical and spinal neurons [17–19]. Temporal genetic ablation studies reveal that GDE2 has roles in adulthood that are distinct from its developmental functions [20, 21]. In the mature nervous system, GDE2 is required for the survival of spinal motor neurons and, in the brain, for the activation of ADAM10 (A Disintegrin and metalloprotease domain-containing protein 10) α-secretase processing of APP via cleavage and inactivation of the GPI-anchored metalloprotease inhibitor RECK (Reversion inducing cysteine rich protein with Kazal motifs) [20, 21]. Notably, GDE2 abnormally accumulates in intracellular compartments in the brains of patients with AD, ALS, and ALS/FTD [21, 22]. Supporting GDE2 dysfunction in disease, amounts of membrane RECK are increased in AD brain, and proteomic studies show a disproportionate reduction of released GPI-anchored proteins in the cerebrospinal fluid (CSF) of patients with ALS compared with controls [21, 22]. These observations raise the possibility that the erosion of GDE2 function may contribute to disease-related cellular pathologies. However, whether the loss of GDE2 affects behavioral outcomes associated with disease or specific brain areas is not known.

Using mice as a model system, we determined the expression pattern of GDE2 in the adult brain and examined the behavioral phenotypes of mice lacking GDE2 (*Gde2*KO). We show here that GDE2 is expressed in brain areas involved in cognition, learning, and memory, as well as motivational aspects of behavior. Behavioral changes can occur early in ADRDs and progress over time with worsening cellular and molecular pathological changes. Since *Gde2*KO mice exhibit neurodegenerative changes by 19 months, we chose to start our behavioral tests prior to this time point to capture behavioral changes that may develop earlier [21, 22]. We conducted several behavioral tasks in WT and *Gde2*KO mice at 7 months of age and explored more complex social and memory phenotypes at 11 and 16 months. We chose to test animals for general locomotor activity, anxiety, sensorimotor reflexes and auditory gaiting, sociability, learning, and memory, because these are the phenotypes that are dysregulated in a variety of mouse models of neurodegenerative diseases. We find that adult *Gde2*KO mice exhibit hyperactivity (7 and 16 months), reduced startle response (7 months), and altered prepulse inhibition (7 months). Aged *Gde2*KO mice also display reduced social motivation (11 months) and impaired short- and long-term memory (16 months), as well as deficits in cued fear memory and secondary contextual fear acquisition (11 months). Overall, our observations show that loss of GDE2 influences diverse behaviors, some of which are manifest in established mouse models of neurodegenerative diseases such as AD and ALS/FTD.

## Methods

### Tissue processing and in situ hybridization via RNAscope

Mice were anesthetized with Avertin solution and perfused transcardially with 0.1 M phosphate buffer, pH7.4 (PB), followed by 4% paraformaldehyde (PFA) in 0.1 M PB as previously described [20]. The brain tissues were harvested, post-fixed in 4% PFA overnight, and then transferred to 30% sucrose at 4C for at least 3 days. The tissues were embedded in O.C.T, flash frozen, and sectioned at 20–30 µm on Leica 3050S.

RNAscope coupled with immunohistochemistry was carried out using the RNAscope Multiplex Fluorescent v2 Assay with the Co-Detection kit according to the manufacturer’s protocol. *Gde2* (*Gdpd5*) mRNA was visualized using the probe Mm-Gdpd5-C2 (ACDBio, Cat No. 494181-C2) and Opal-570 fluorophore (Akoya Biosciences, FP1488001KT, 1:500), and costained with NEUN (Millipore Sigma, MAB377, 1:500). Slides were mounted with ProLong™ Gold Antifade Mountant with DAPI (ThermoFisher, P36931), coverslipped, and imaged on confocal microscope Zeiss LSM800.

## Behavioral experiments

### Subjects

All procedures involving animals were performed in accordance with the guidelines of the Johns Hopkins University Institutional Animal Care and Use Committee. Male and female *Gde2*KO and WT mice on a C57BL/6J strain background were used for all tests and were genotyped and maintained as previously described [18]. To obtain these mice, breeding cages were set up with 1 male and 2 female mice, all three of which were either *Gde2*KO or WT, so that the pups in each litter were all one genotype. This breeding scheme was utilized in order to generate a large number of animals (> 70) in a short period of time to obtain the appropriate cohort sizes. To minimize variation, all animals were of the same genetic background and generated from the same number of crosses. Two cohorts of 30–40 mice, 1.5 months apart in age, with approximately equal numbers of *Gde2*KO and WT mice were tested in the same sequence of behavioral experiments. Results from both cohorts were pooled together for analysis for all tests. Mice were kept in cages where up to 5 males and females were housed together separated by sex. All mice were inspected before any behavioral tests were conducted and any that displayed injuries or health problems were removed from the cohort. Mice were housed in a 12-h light/dark cycle, where mice were tested at the start of the light cycle in rooms and chambers in which the light was turned on and sound disturbances were minimized.

All tests were performed by DD and AW in isolated behavior rooms with ambient temperatures with low background noise levels. Mice were handled daily for 3 days before the start of all behavioral testing. Testing was conducted during the light phase of the circadian cycle, with experimenters blinded to genotype while testing was performed. Before every test, mice were transferred to the testing room to habituate to the new location for at least 30 min. Behavioral performances in the Open Field (OF), Y maze, plus maze, Fear Conditioning (FC), Social Motivation, and Morris Water Maze (MWM) tests were recorded by a computer-based video tracking system (ANY-maze, Stoelting Co., IL). For any tests where unexpected noises could cause startle responses and disrupt the tests, gentle lo-fi music was played to provide quiet background sound. PPI of acoustic startle reaction (ASR) was conducted in a startle soundproof chamber (model SR-LAB, San-Diego Instruments, CA). In the two tests that were performed at two different ages (OF and Y maze), conditions were altered slightly between testing ages; specifically, we utilized testing apparatuses that were constructed of different materials and changed the room in which the tests were conducted to prevent initial testing memories from interfering with the performance of the mice. Since no learning or memory deficits were observed in tests conducted at the 7-month time point and Fear Conditioning and Morris Water Maze are stressful tests for animals, we conducted these tests at a single time point (11- and 16-months, respectively). The 5-month gap was to allow the mice sufficient recovery time before embarking upon the Morris Water Maze test. For tests that were conducted at the same age point, at least 5 rest days were given to mice between different tasks to allow for sufficient recovery from previous exposure to novel environment and equipment.

### Open field: locomotor activation and anxiety measurement task

Locomotor activity and anxiety measurements in Open Field (OF) experiments were assessed in two different settings depending on the age of the mice using similar equipment and protocols as previously described [23]. At the 7-month time point, mice were placed in a clear plexiglass OF arena box (40 cm by 40 cm by 40 cm) for 30 min with the lights off. At the 16-month time point, mice were placed in a different OF arena box made of white plastic (37 cm by 37 cm by 35 cm) for 45 min with the lights turned on. Before each trial, the cages were rubbed with bedding from the home cage, cleaned with a 30% ethanol solution, and dried with a paper towel. The trial began with the mouse being released near the OF wall. The total distance traveled during each block of 5 min, along with the distance and percentage of time spent in the center vs perimeter of the box as measures of anxiety-like behavior, were analyzed. The percent of time spent in the center area due to chance was 34%.

### Y maze: novelty-induced locomotor activation and spontaneous alternation task

Mice were tested in a Y-shaped maze using a similar protocol as previously described [24]. Each maze arm measures 7 cm by 48 cm. For the 7-month test, a wooden Y maze was used, while the 16-month test was conducted in a different behavioral room with a metal Y maze. The different room, maze color and texture, and spatial cues were all used to prevent similarities between the tasks. Both mazes were elevated 70 cm off the ground. Before each mouse was tested, the maze was rubbed with bedding from the home cage, cleaned with 30% ethanol solution, and dried. In trial 1, mice were placed in one end of the start arm while one of the remaining two arms was blocked off. The arm selected to be blocked off alternated for each mouse tested. All mice explored the maze freely for 5 min. The number of arm entries and locomotor activity were recorded using ANY-maze software (Stoelting Co., IL). Trial 2 began 30 min after the initial exploration period. The arm block was removed, and mice were placed at the end of the start arm and allowed to explore all 3 arms for 5 min. The time spent in the novel arm compared to the previously explored arm in the second trial and the total distance traveled in both trials were analyzed using the ANY-maze software.

### Plus maze: hyperactivity and anxiety assessment task

Mice were allowed to run freely for 10 min in an elevated plus maze to assess motor activity in this environment and evaluate how much time the mice spent in the closed arms vs. open arms as a metric of anxiety-like behavior using similar equipment and protocols as previously described by Savonenko et. al. [25]. The plus maze consisted of 2 open arms and 2 closed arms (all measuring 50 cm × 10 cm) extending from an open square platform (10 cm × 10 cm). All arms were made of wood with plastic walls (40 cm high) for the closed arms and were angled at 90 degrees to each other (see Fig. 2K). Like the Y maze, the plus maze was elevated on 70 cm tall metal poles. Using ANY-maze software, the total distance traveled by the mice and the time spent in the open and closed arms were tracked. Before starting the trials, the maze was wiped down with bedding. Before each trial started, the maze and walls were wiped down with a 30% ethanol solution and dried with disposable paper towels.

### Social motivation: 3 chamber social preference task

The social preferences of mice were tested in a three-chamber apparatus using similar protocols and equipment as previously described [26]. The apparatus was constructed from opaque Plexiglas (60 cm long × 40 cm wide × 35 cm high) with 2 middle dividers with cutout doors dividing the chamber into 3 equal-sized compartments (see Fig. 4A). During the 5-min habituation trial, mice were allowed to roam freely in the compartments. In both side chambers, there was one inverted empty mesh metal cup holder. After a 30-min delay in which the test mouse was removed to a waiting cage, the mouse was put back in the middle compartment for the social motivation trial and again allowed to explore for 5 min. A 2-month-old male WT mouse on a C57BL/6J background was used as the stimulus animal. This mouse came from a set of three littermates that were alternated between each test. The stimulus animal was placed under the mesh cup holder in one side chamber of the apparatus, while a blue ball toy was placed under the cup holder in the other side chamber. The distance traveled in each compartment, as well as the amount of time spent in the areas directly around the mesh enclosures was analyzed using ANY-maze. The area around the mesh was 7 cm wide circle as defined only in the AnyMaze program, no physical circle was present in the cage. This size annulus was based on the tracking of the nose and central point of the mouse body while they investigated the meshed enclosure with the social object. After each trial, all compartments and the metal cup holders were cleaned with a 30% ethanol solution and dried afterward.

### Prepulse inhibition: startle reflex and prepulse inhibition task

Testing for the prepulse inhibition (PPI) task was conducted using similar protocols and equipment as described previously by Savonenko et. al. [25]. Briefly, mice were tested in a closed off box equipped with speakers while the lights inside were turned off (29 cm wide × 30 cm deep × 29 cm high). The mice were placed in a small cylinder in the center of the box that sat on sensors that could record the latency and maximal amplitudes of the startle response when the mice reacted to the pulse stimuli or pre-pulse (SR-LAB Startle Response System, SIC 002668). After a 5-min acclimation period, the mice were exposed to 10 startle pulses (25 ms, 120 dB) of white noise. This was used to determine the ASR (Acoustic Startle Reaction) at the beginning of the test. Mice were then given 52 trials of either prepulse-pulse stimuli, pulse stimuli, or background-only noise in a pseudorandom pattern every 30 s to measure the startle reaction as the test progressed and the PPI at different prepulse levels. Prepulse levels were 25 ms pulses of white noise, slightly higher than background noise (63 dB) levels. 6 trials each for the following prepulses were given and averaged together for analysis: 74, 78, 82, 86, and 90 dB. Prepulses were delivered 75 ms before the startle pulse was delivered. The maximum startle amplitude and latency for the pulse delivery were analyzed and used to calculate the PPI in prepulse trials and ASR in pulse-only trials (see Fig. 3G). PPI was calculated according to the following formula: % PPI = 100 × (reactivity on pulse alone trials – reactivity on prepulse trials + pulse trials)/reactivity on pulse alone trials.

### Morris water maze (MWM): short and long-term spatial memory task

The MWM task was performed using similar equipment and protocols as described previously by Savonenko et. al. [27]. Briefly, mice were trained to swim in a large water pool (130 cm diameter) with a clearly delineated swimming path. The water was kept at 21 ± 1 ℃ and colored with non-toxic washable white paint to make the platform invisible. Visual cues were placed around the edge of the maze and along the walls of the training room. All mice were pre-trained for 4 days to build stamina and learn to swim and stay on submerged platform (~ 1 cm below the surface). The purpose of the pretraining was for the mice to learn the procedural aspects of the MWM before the training phase. Mice were allowed to swim until they found the platform or until 60 s elapsed, at which point they were directed to the submerged platform and then removed to a warm, dry holding cage after sitting there stably for 5 s. The training phase to test spatial memory started in a different room with a set of big visual cues situated along the walls (distal cues) and a set of smaller visual cues situated on a rim of the pool (proximal cues). During this phase, mice were tested a total of 6 times a day for 4 days. During the first two training days, the platform was placed in quadrant 1, and the mice were started at designated positions in the other 3 quadrants in a semi-random order and varied for each trial (Fig. 5A). At the next reversal stage which lasted for 2 training days, the platform was moved to quadrant 3, and the mice were started randomly at positions in quadrants 2 and 4. During each day, two types of trials were conducted (Fig. 5A): training trial in which the platform was hidden under the water and mice were able to climb on it as soon as they reached it. The second type of trials, called probe trials, were conducted with the platform collapsed at the bottom for the duration of the probe trial. If the mice could not find the platform 20 s after the platform was raised, they were directed to the platform as previously described for training trials. The probe trials lasted 40 s after which the platform was raised to allow the animal to climb on it. To test the short- and long-term memory of the platform location, the probe trials were conducted at the end and at the beginning of a training day, respectively. Short-delay probe trials were run ~ 20 min after the last training trial, and the long-delay probe trials were conducted after the over-night delay (~ 20 h). Probe tests were given at the end of each day to assess the short-term memory of platform location from the previous 5 trials the mice had just completed that day. Probe tests at the beginning of days 2–4 were given to assess the long-term memory the mice had of the platform location from the previous day. These types of probe trials were used in numerous previous studies of cognitive deficits in different mouse and rat models of Alzheimer’s and other diseases [23, 27–29]. The amount of time spent around the quadrant platform locations (40 cm diameter circle) was analyzed using ANY-maze software, along with swim distances and latency to find the platform for each trial.

### Fear conditioning (FC): delayed Pavlovian fear acquisition and memory task

The FC task uses classical conditioning to train mice to associate a neutral conditioned stimulus (CS, audio tone) and novel context with an aversive unconditioned stimulus (US, foot shock) and we performed this task using similar protocols and equipment as previously described [30]. After training, mice learn to associate the CS and context with the US and exhibit a freezing response, an unconditioned fear response when presented with the CS or the same context. A mouse training chamber with a metal grated bottom that delivered the foot shock (Stoelting Co., Wood Dale, IL) was used (17 cm wide × 17 cm long × 25 cm high) (Fig. 6A). With lights turned on inside the chamber, mice were released into the box and allowed to roam freely. After 120 s (intertrial interval, ITI), a 15-s long 85 dB, 600 Hz tone (CS) was delivered, followed immediately by a 2-s long 0.6 mA foot shock. This was performed 3 times, followed by one last ITI. Freezing durations of longer than 1 s were measured using ANY-maze software. The cage was cleaned with a 30% ethanol solution at the start and end of each test. 24 h after trial 1, mice were tested in the same context for 5 min with no CS or US to test for contextual fear memory (trial 2). 3 h after trial 2, the test chamber was substituted for a different shape, size, and color box (25 cm wide × 25 cm long × 38 cm high with curved white paper insert in one corner, all white walls, and solid floor) in which small amounts of bedding from the home cage were scattered. Mice were tested using the same paradigm as in trial 1 without the US (only CS). The percent time spent freezing in all contexts was measured during the ITI and during the CS.

### Statistics

Statistics were analyzed using Statistica 13.3 (TIBCO Software Inc., CA) with a minimum significance level of *P* < 0.05. Two-way ANOVA tests were used for a majority of the behavioral experiments, after which Fisher’s least significant difference post hoc tests were conducted to examine significant main effects/interactions. Genotype and sex were treated as the main effects, while effects of time intervals, area, trial type, arm type, or object type were treated as repeated measures. Interactions were orthogonal sets. Bonferroni corrections were performed on individual comparisons when the main effect was not significant. See Additional file 2: Table S1 for a detailed list of tests run. The number and sex of all animals are included in the main and supplemental figures. The means were all graphed with error bars that represent the SEM.

## Results

### GDE2 is expressed in the adult mouse brain

**Fig.1:**
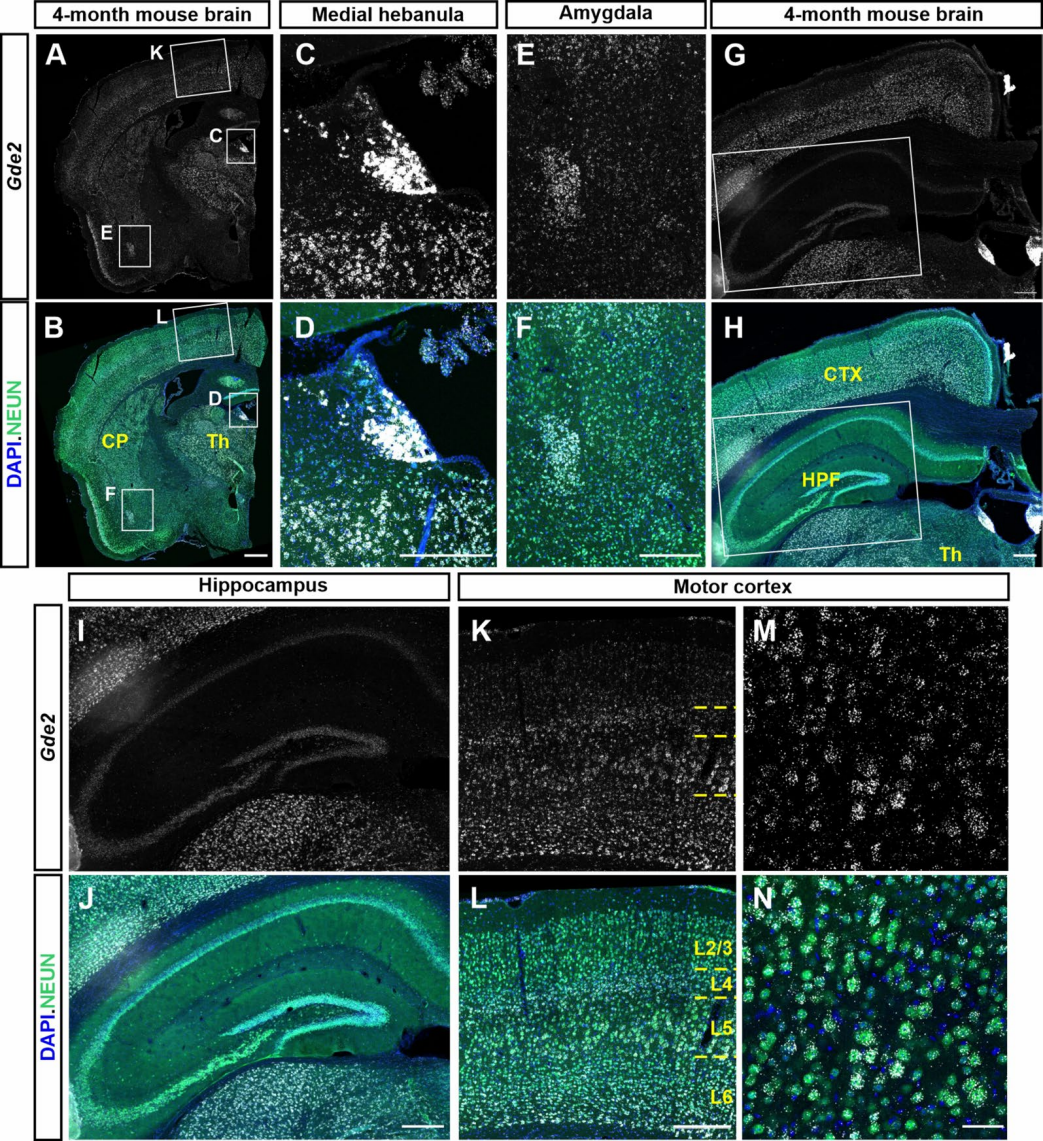
*Gde2* mRNA expression in the adult brain. **A**–**N** Exemplar images of RNAscope detection of *Gde2* transcripts (white) in the adult brain. Nuclei are marked by DAPI staining (blue), and NeuN antibody staining (green) marks neurons. Boxed areas in **A** and **B** are magnified in panels (**C**, **E**, **K**) and (**D**, **F**, **L**), respectively. Boxed areas in **G** and **H** are magnified in panels **I**, **J**. Hatched lines in **K** and **L** mark cortical layers. Scale bar: (**A**, **B**): 500 µm; (**C**–**L**): 250 µm; (**M**, **N**): 50 µm. CP: Caudate Putamen, Th: Thalamus, CTX: Cortex, HPF: Hippocampal Formation

GDE2 is expressed in the developing and postnatal spinal cord and brain [20, 31, 32]; however, its expression in the adult brain has not been determined. To assess GDE2 expression in the adult mouse brain, we performed RNAscope for *Gde2* transcripts in coronal sections from 4-month-old WT mice. We detected *Gde2* expression in major brain areas including the thalamus (Th) and caudate putamen (CP) (Fig. 1A, B), with particularly strong expression in the medial habenula and the medial and anterior amygdala (Fig. 1C–F), which are areas associated with regulation of emotional and motivational aspects of behavior including fear/anxiety responses [33, 34]. *Gde2* is also expressed throughout the hippocampus, a region implicated in learning and memory (Fig. 1G–J) [35, 36]. In addition, *Gde2* transcripts are detected in the cortex with enriched expression in deep cortical layers 4–6 (Fig. 1K, L). Similar to previous studies using younger mice [31, 32], *Gde2* expression in the cortex is mainly localized to neurons with some expression in non-neuronal cells (Fig. 1M, N). This aligns with previous expression and transcriptomic data showing *Gde2* expression in neurons, mature oligodendrocytes, and endothelial cells [37, 38].

### General health of *Gde2*KO mice

Similar to previously published work [18], *Gde2*KO mice were viable, fertile, and born at the expected Mendelian frequencies. Male and female *Gde2*KO mice were born in about equal numbers. *Gde2*KO mice showed no obvious differences in health or appearance from WT mice. However, upon weighing, male *Gde2*KO mice consistently have slightly lower body weight compared to WT mice (~ 10%, Additional file 1: Fig. S1A). This difference in body weight between genotypes decreases with age (Additional file 1: Fig. S1A). No difference in body weight was observed for female mice (Additional file 1: Fig. S1B).

### *Gde2KO* mice exhibit hyperactivity

**Fig. 2:**
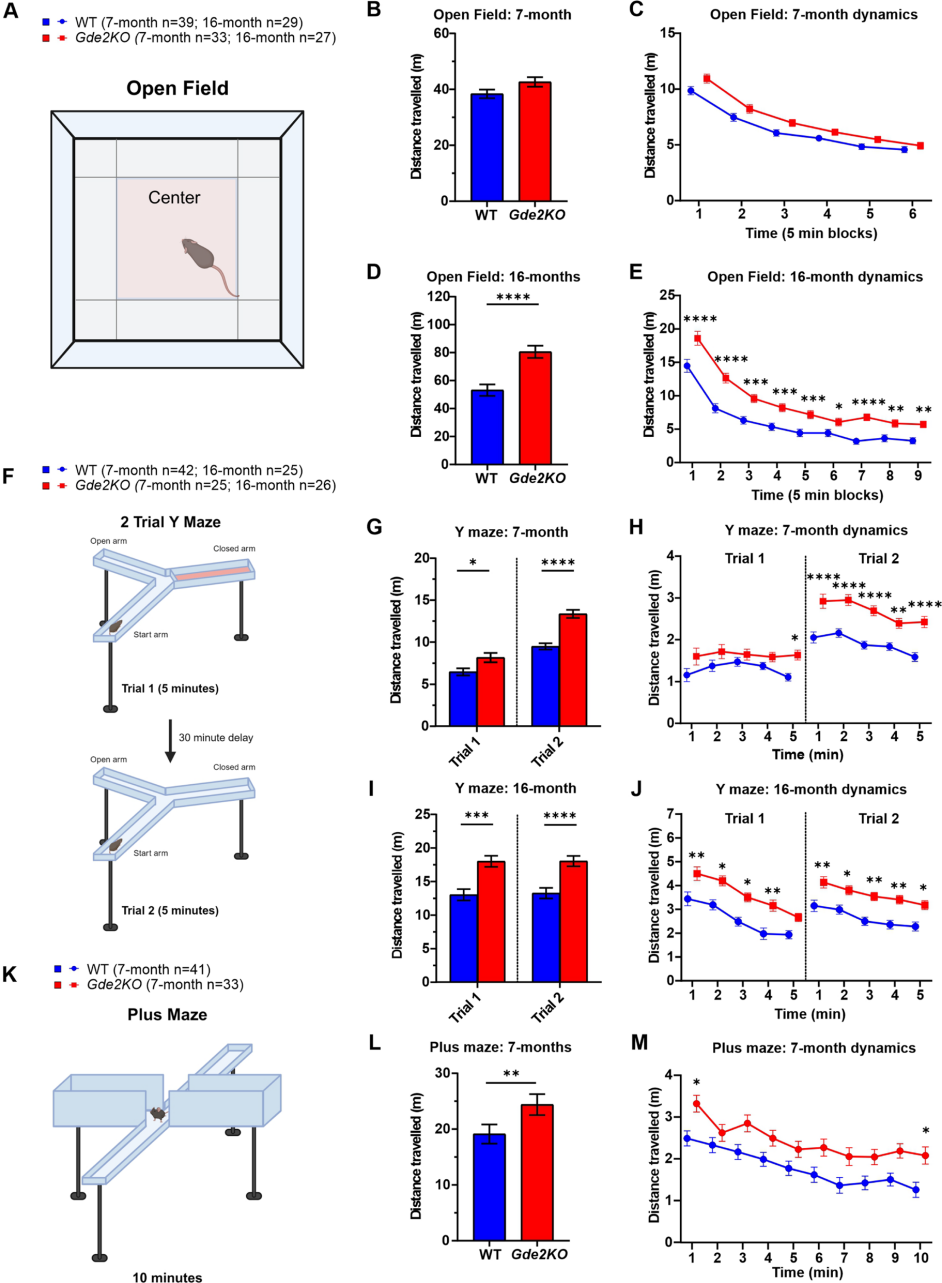
Hyperactivity phenotype in *Gde2*KO mice. **A** Schematic of OF test. **B**–**E** Analysis of OF test. **B**, **C** Total distance (**B**) and its dynamics (**C**) during 30 min of testing in the OF task for WT and *Gde2*KO mice at 7 month time point. No effect of genotype or its interactions were detected (three-way mixed design ANOVA, Ps > 0.05). **D**, **E** The same measures and analysis as in B and C, respectively, for 16-month-old mice. *Gde2*KO mice demonstrated higher motor activity that was partially ameliorated during the duration of testing as indicated by significant effect of genotype (F(1,52) = 20.92, P < 3.0E−5) and genotype × block interaction (F(8,416) = 2.15, P < 0.031). **F** Schematic of 2 trial Y maze test. **G**–**J** Analysis of Y maze task (**G**, **H**). Total distance (**G**) and dynamics (**H**) during Y maze testing in 7-month-old mice. *Gde2*KO mice were more active than WT mice during trial 1 (one arm closed) (ANOVA, effect of genotype F(1,63) = 5.95, P < 0.018) and particularly during trial 2 (all 3 arms open) (F(1,63) = 39.97, P < 1.0E-5). **I**, **J** The same measures and analyses as in G and H, respectively, for 16-month old mice. *Gde2*KO mice were more active than WT mice in both trials (F(1,47) > 17.75, P < 1.0E−4). **K** Schematic of plus maze. **L** The distance traveled (**L**) and its dynamics (**M**) by 7-month-old WT and *Gde2*KO mice. The higher motor activity of *Gde2*KO mice was confirmed by ANOVA (effect of genotype, (F(1,70) = 10.35, P < 0.002). All graphs are means ± SEM; *P* > 0.05; *P < 0.05, **P < 0.01, ***P < 0.001, and ****P < 0.0001. See Additional file 2: Table S1 for statistical details. Schematics in **A**, **F**, and **K** created in BioRender.com

Animals were first tested in the Open Field (OF) task at 7-months of age (Additional file 1: Fig. S1C). At the 7-month time point, WT and *Gde2*KO mice showed no significant difference in the distance traveled in the OF chamber (Fig. 2A–C, Additional file 1: Fig. S2A–C). We next tested these same mice at 16-months of age to see if any age-progressive phenotypes emerged (Additional file 1: Fig. S1C). At this time point, *Gde2*KO mice showed significantly increased locomotion, and this hyperactivity was the most pronounced at the beginning of testing (Fig. 2D, E). Importantly, despite increased novelty-induced activity, *Gde2*KO males demonstrated significant habituation as testing progressed (Additional file 1: Fig. S2D, E). Female *Gde2*KOs showed a trending increase in activity at both ages; however, these differences did not reach significance (Additional file 1: Fig. S2A, C, D, F).

Hyperactivity in *Gde2*KO mice was also evident in the two-trial Y maze at 7- and 16-months of age and in both sexes (Fig. 2F–J, Additional file 1: Fig. S2G–L). In this task, mice explore a Y-shaped maze across two 5-min trials. In the first trial, they are allowed to explore two of the arms. In the second trial, a novel third arm is opened for exploration (Fig. 2F, see “Methods”). Notably, at 7-months of age, the between-genotype differences in distance traveled were more pronounced in trial 2 than in trial 1, indicating increased hyperactivity in *Gde2*KOs upon the opening of the new arm in the second trial (Fig. 2G, H). At 16-months of age, increased activity of *Gde2*KO mice was observed in trial 1 and did not increase further in trial 2 (Fig. 2I, J).

In the plus maze task, *Gde2*KO animals at 7-months were tested to see if hyperactivity was novelty induced (Additional file 1: Fig. S1C). Mice were placed in a plus shaped maze with open and closed arms and were allowed to explore for 10 min (Fig. 2K; see “Methods”). Compared to WT, *Gde2*KO mice travel more distance over time compared with WT in both open and closed arms (Fig. 2L, M). The between-genotype difference is observed in both males and females (Additional file 1: Fig. S3A–C). Notably, we see a significant difference in distance traveled by *Gde2*KOs during the first minute of the test when the environment is most novel (Fig. 2M). We further analyzed the distance traveled by mice in the plus maze task in blocks of 5 min to match the block durations of the OF analysis. *Gde2*KOs still demonstrate hyperactivity when analyzed in 5-min blocks of time (Additional file 1: Fig. S3D). This observation is in agreement with the hyperactivity phenotypes observed in the Y maze task at 7 months.

Taken together, *Gde2*KO mice display hyperactivity at younger ages mainly in response to new situations/environments; however, aged *Gde2*KO mice show hyperactivity across all tasks measuring distance traveled regardless of novelty. Importantly, despite the hyperactivity observed in *Gde2*KO mice, the extent of the hyperactivity was moderate and not debilitating as the motor exploration in these mice showed preserved habituation across all tests.

### Normal anxiety behavior in* Gde2*KO mice

**Fig. 3:**
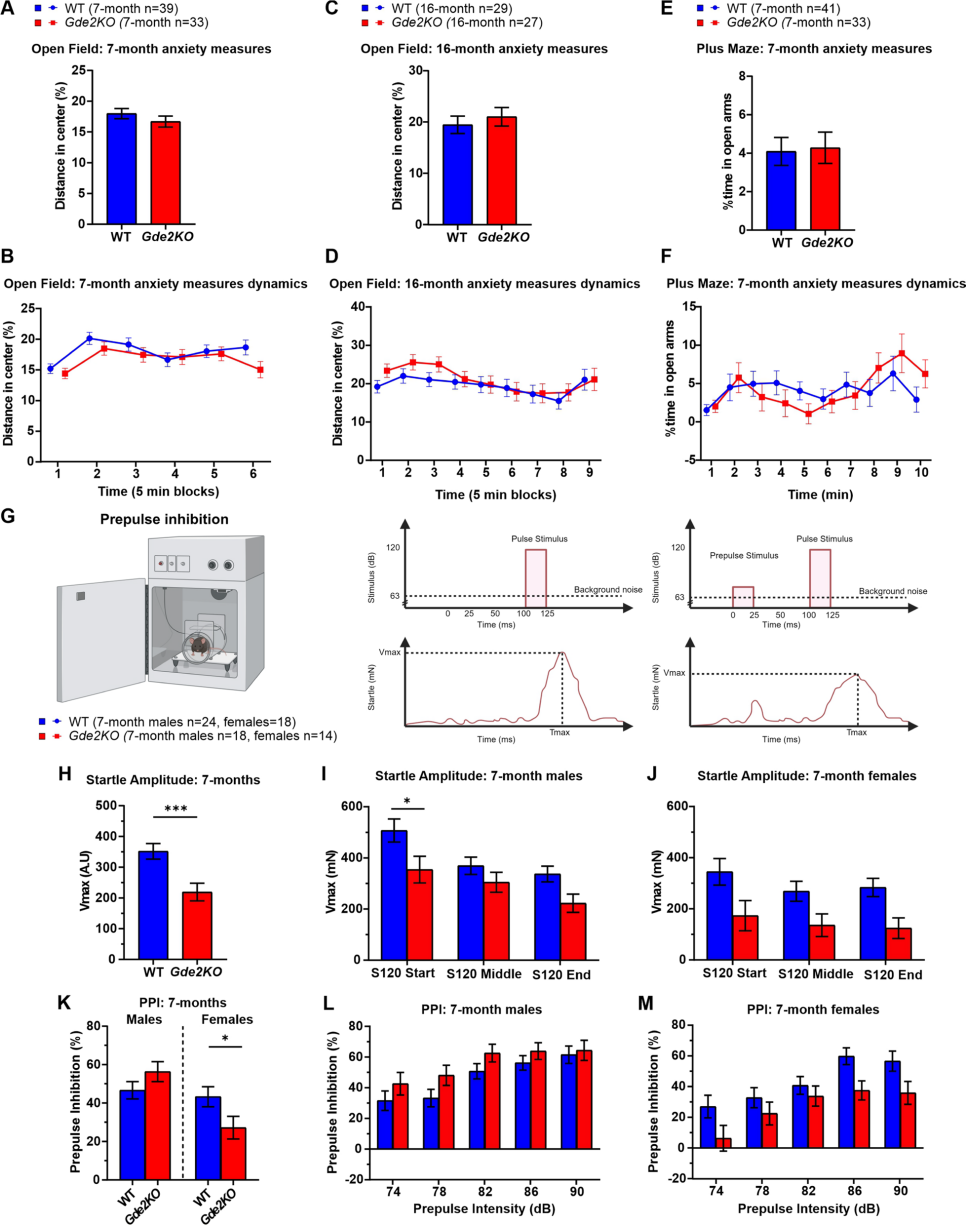
Anxiety, startle response, and PPI assessment in *Gde2*KO mice. **A**–**D** Analysis of anxiety phenotype in OF test. **A**, **B** Total percent distance traveled in the center of OF setup (**A**) and its dynamics (**B**) at 7 months. No effect of genotype or its interactions were detected (three-way mixed design ANOVA, P > 0.05). **C**, **D** The same measurements and analysis as in A and B, respectively, for 16-month-old mice (ANOVA, P > 0.05). **E**, **F** Total percent distance traveled in the open arms of the plus maze (**E**) and its dynamics (**F**) at 7 months. No effect of genotype or its interactions were detected (ANOVA, Ps > 0.05). **G** Schematic of PPI task setup (left) and illustration of prepulse and pulse stimulus delivery above the corresponding startle response (middle and right). **H**–**J** Combined startle response (**H**) and the dynamics separated by males (**I**) and females (**J**) when the 120 dB pulse stimulus was delivered. *Gde2*KO mice had lower startle amplitude than WT mice (ANOVA, effect of genotype F(1,70) = 12.02, P < 0.0009). Male *Gde2*KO mice showed the most notable startle response decrease at the start of the test (Fisher LSD post-hoc P < 0.05). (K-M) Mean %PPI for all prepulse levels (**K**) and %PPI at each prepulse stimulus intensity are shown for males (**L**) and females (**M**). The three-way mixed design ANOVA test revealed a significant effect of genotype × sex interaction (F(1,70) = 6.09, P < 0.02), with female *Gde2*KO mice having significantly lower %PPI (Fisher LSD post-hoc P < 0.05). All graphs are means ± SEM; ns, *P* > 0.05; *P < 0.05, and ***P < 0.001. See Additional file 2: Table S1 for statistical details. Schematic in **G** created in BioRender.com

Mice normally avoid open spaces such as the center area of the OF and the open arms in the plus maze. Thus, distance traveled and time spent in these areas can be used as a measure of anxiety levels [39, 40]. *Gde2*KO mice were tested at 7 months and 16 months in the OF test to determine if they had an age-progressive anxiety phenotype. At both time points, male and female *Gde2*KO mice show no difference in the percent distance traveled in the center of the OF arena (Fig. 3A–D; Additional file 1: Fig. S4A–F). Additionally, no difference in the percent time spent in the center was found between genotypes at either age (Additional file 1: Fig. S4G–K). In line with these observations, WT and *Gde2*KO mice spent an equivalent amount of time in the open arms of the plus maze (Fig. 3E, F) and showed no significant difference in distance traveled in the open arms whether analyzed in 1- or 5-min blocks of time (Additional file 1: Fig. S4L–N). Since *Gde2*KO mice in the OF task showed no increased anxiety phenotype between time points, the plus maze task was not repeated at the later age. In sum, these observations suggest that loss of GDE2 has no effect on anxiety levels.

### *Gde2*KO mice show differences in startle response and PPI

Startle response and sensorimotor gating can be assessed using the startle reflex and pre-pulse inhibition (PPI) to acoustic stimuli. In this test, animals exhibit a startle reflex in response to a sound stimulus (pulse). Their startle reflex is typically dampened by playing a lesser prepulse ahead of the main pulse. This dampening of the startle response is indicative of sensorimotor gating [41].

To test if loss of GDE2 affects these behaviors, we placed 7-month-old mice in a startle chamber and played a series of pulses or pulses preceded by a prepulse (Fig. 3G; Additional file 1: Fig. S1C; see “Methods”). *Gde2*KO mice exhibit a significantly reduced startle amplitude in response to the sound stimulus (Fig. 3H). The most significant difference in startle response for male *Gde2*KOs was at the start of the test and was less pronounced when the startle amplitude decreased due to habituation (Fig. 3I). *Gde2KO* females show a significant decrease in startle response (Additional file 1: Fig. S5A) that was not modulated by habituation (Fig. 3J, trending decrease not significant after Bonferroni correction).

No overall differences in PPI were observed between WT and *Gde2*KO mice (Additional file 1: Fig S5B); however, when analyzed separately for each sex, there was a significant reduction in PPI for female *Gde2*KO mice (Fig. 3K). This reduction in PPI for females was observed as a main effect of genotype without any interaction of genotype and level of prepulse (Fig. 3M). Male *Gde2*KO mice do not exhibit any changes in PPI (Fig. 3K, L). We examined the correlation between startle activity and PPI to see if the difference in startle response between genotypes could be related to the levels of PPI. These phenotypes appear independent, with startle reactivity accounting for less than 36% of the variability in PPI in all cases (Additional file 1: Fig. S5C). Since a startle and PPI phenotype was evident at the 7-month time point in *Gde2*KO mice, we did not retest the animals in this task at a later time point. Based on these observations, we conclude that *Gde2*KO mice of both sexes have reduced startle reactivity, with female *Gde2*KOs showing additional deficits in PPI.

### Aged *Gde2KO* mice show differences in social motivation and spatial working memory

**Fig. 4:**
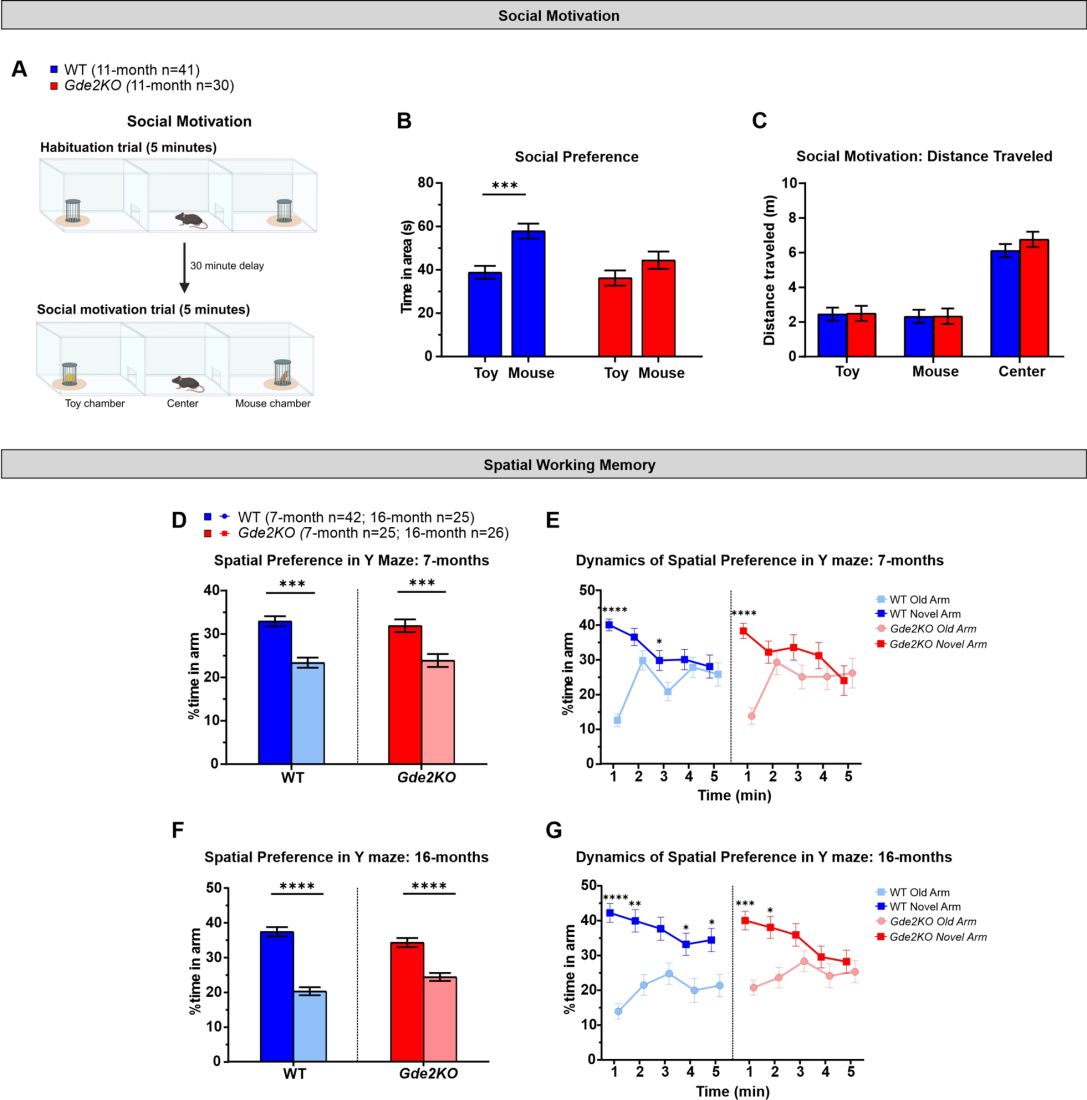
*Gde2*KO mice exhibit social motivation and spatial working memory deficits. **A** Schematic of social motivation test. The stimulus mouse and toy are only present during the social motivation trial. **B** Total time spent in mouse and toy areas during social motivation trial at 11 months. A significant effect of genotype is seen (three-way mixed design ANOVA, F(1,67) = 5.58, P < 0.022), with WT mice showing an increase in time spent by the mouse area compared to the toy area (Fisher LSD post-hoc P < 0.0003) while *Gde2*KO mice showed no difference in time spent between the two areas (Fisher LSD post-hoc P > 0.05). **C** Total distance traveled during the duration of the social motivation trial in each compartment. No effect of genotype or its interactions were detected (ANOVA, Ps > 0.05). **D**–**G** Y Maze spatial memory analysis. **D**, **E** Total percent time spent in novel vs. new arm during trial 2 (**D**) and dynamics at 7 months. WT and *Gde2*KO mice spent significantly more time in the novel arm compared to the old arm (ANOVA, effect of arm, F(1, ≥ 23) > 18.58, P < 0.0003) with the largest difference during the first minute. **F**, **G** The same measures and analyses as in **D** and **E**, respectively, for 16-month-old mice. Again, WT and *Gde2*KO mice spent significantly more time in the novel arm compared to the old arm (ANOVA, effect of arm, F(1, ≥ 23) > 38.95, P < 0.0001). WT mice show a preference for the novel arm throughout the 5-min trial while *Gde2*KO mice only show a preference for the novel arm at the start. All graphs are means ± SEM; ns, *P* > 0.05; *P < 0.05, **P < 0.01, ***P < 0.001, and ****P < 0.0001. See Additional file 2: Table S1 for statistical details. Schematic in **A** created in BioRender.com

GDE2 is expressed at high levels in the medial habenula (Fig. 1C, D), an area associated with encoding social behaviors [33]. Accordingly, we used the 3-chamber social preference test to evaluate social motivation in the absence of GDE2 (Fig. 4A). Briefly, one chamber contained a wire enclosure with a stimulus mouse, and the other chamber contained a wire enclosure with a toy. After a habituation trial where both chambers were empty, the mouse being tested was placed in the middle chamber, and social preference was assessed based on time spent around the stimulus mouse compared to the toy (Fig. 4A; see “Methods”). Mice were tested at 11-months in the social motivation task (Additional file 1: Fig. S1C, see “Methods”). While WT mice spent significantly more time with the mouse than the toy, *Gde2*KOs showed no preference between the two (Fig. 4B, Additional file 1: Fig. S6A). This suggests that *Gde2*KO mice have reduced social preference compared to WT animals. This loss of social preference is seen in both male and female *Gde2*KO mice (Additional file 1: Fig. S6B–E). During the social motivation trial, *Gde2*KOs showed no differences in motor activity that could confound interpretation (Fig. 4C). However, during habituation, there was an increase in the activity of *Gde2*KO mice, particularly females, in the center compartment but not in the other two compartments (Additional file 1: Fig. S6F–J). Since *Gde2*KO mice exhibited a decrease in social preference at the 11-month time point, we did not repeat the task at 16 months.

We further tested the spatial working memory of *Gde2*KO mice using the two-trial Y maze described earlier (Fig. 2F; see “Methods”). In this paradigm, the time spent in the novel arm in trial 2 is indicative of spatial working memory. At 7-months, both WT and *Gde2*KO mice prefer to spend time in the novel arm rather than the old arm (Fig. 4D, Additional file 1: Fig. S1C). As is typical, both genotypes show a strong preference for the novel arm during the first minute of the test (Fig. 4E), and these effects were not modified by sex (Additional file 1: Fig. S7A–D). We tested the mice again at 16-months, since memory deficits can be an age progressive phenotype (Additional file 1: Fig. S1C). WT and *Gde2*KO mice still prefer the novel arm over the old arm on average (Fig. 4F). WT mice retain their preference for the novel arm throughout the task; however, *Gde2*KO mice lose this preference after the first 2 min (Fig. 4G). These results suggest that 16-month-old *Gde2*KO mice have impaired performance in this spatial working memory test compared to WT mice of the same age. These deficits were also observed when males and females were analyzed separately (Additional file 1: Fig. S7E–H). Taken together, aged *Gde2*KO mice show abnormal social and spatial preferences in the time scales associated with working and/or short-term memory. Accordingly, we performed further tests to assess memory and cognition in *Gde2*KO mice.

### Short- and long-term spatial memory impairment in *Gde2*KO males in the Morris water maze

**Fig. 5:**
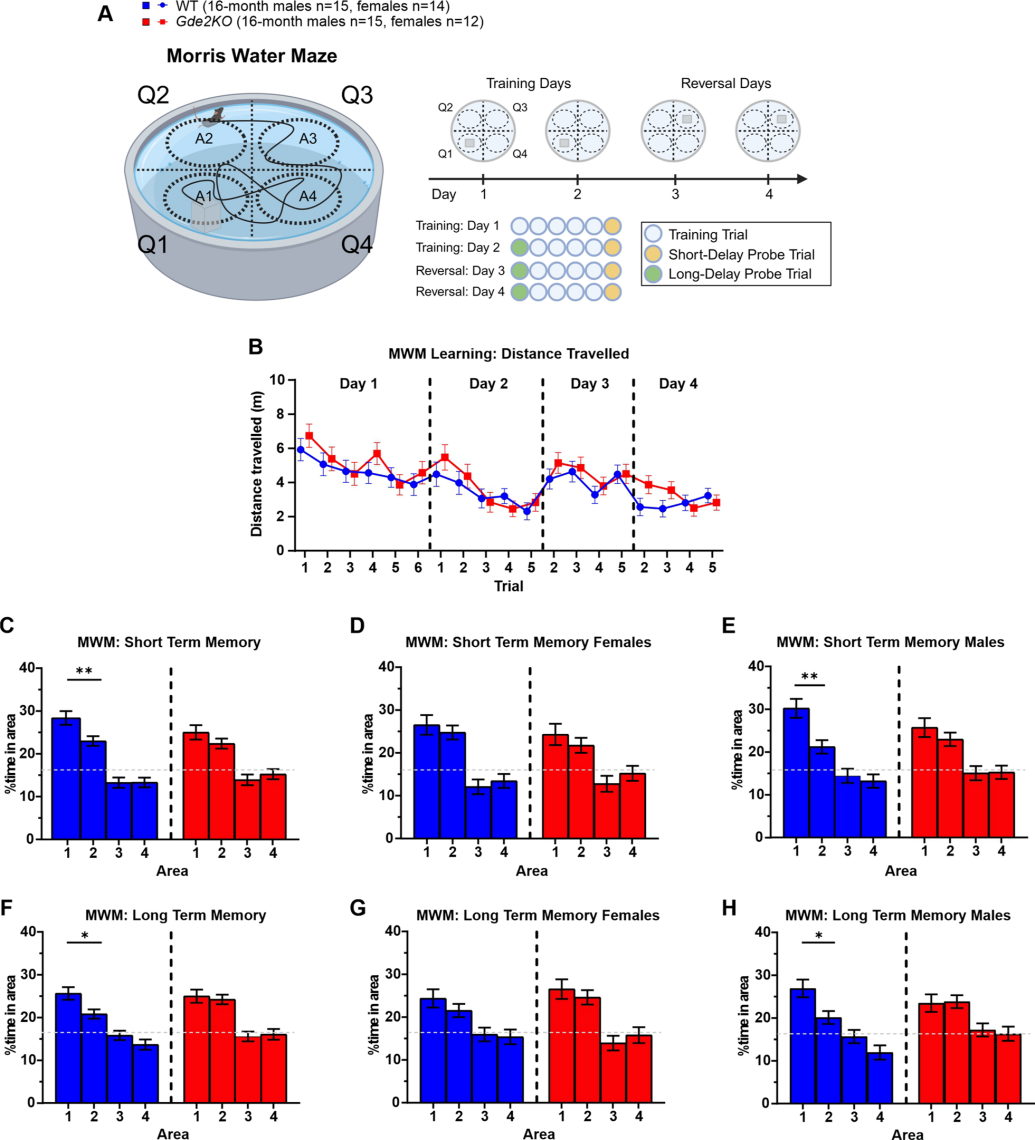
Spatial memory deficits in MWM for *Gde2*KO male mice. **A** Schematic of MWM task. Mice were tested over 4 days: during the first 2 days, the platform was in quadrant 1. During the last 2 days, the platform was moved to quadrant 3. **B** Total distance mice traveled before reaching the platform during non-probe trials. No effect of genotype or its interactions were detected (three-way mixed design ANOVA, Ps > 0.05) (**C**–**E**) Analysis of percent time spent in the central area of each quadrant during the probe trial at the end of day 2. WT and *Gde2*KO mice exhibit differences in time spent across quadrant areas (**C**, ANOVA, effect of area, F(3, ≥ 78) > 15.19, P < 0.0001). However, WT mice and not *Gde2*KO mice spent significantly more time in quadrant 1 compared to quadrant 2 (Fisher LSD post-hoc, P < 0.009 and P > 0.05, respectively). Only male WT mice spent significantly more time in quadrant 1 compared to quadrant 2 (**E**, Fisher LSD post-hoc, P < 0.006), while female WT mice showed no preference between quadrants (**D**, Fisher LSD post-hoc, P > 0.05). **F**–**H** The same measures and analyses as in **C**–**E**, respectively, for the probe trial at the start of day 3. WT and *Gde2*KO mice showed differences in time spent across quadrant areas (**F**, ANOVA, effect of area F(3,78) > 12.91, P < 0.0001). Only WT mice spent significantly more time in quadrant 1 compared to quadrant 2 (Fisher LSD post-hoc, P < 0.024). Specifically, only male WT mice spent significantly more time in quadrant 1 compared to quadrant 2 (**H**, Fisher LSD post-hoc, P < 0.039), while female WT mice showed no preference between quadrants (**G**, Fisher LSD post-hoc, P > 0.05). The dotted lines at 16% represent the expected time spent in each area due to chance. All graphs are means ± SEM; ns, *P* > 0.05; *P < 0.05, and **P < 0.01. See Additional file 2: Table S1 for statistical details. Schematic in **A** created in BioRender.com

The Morris Water Maze (MWM) test was used to assess learning and spatial memory in *Gde2*KO mice [42, 43]. In this task, mice were made to swim in a pool that contained a hidden platform in one quadrant (Fig. 5A; see “Methods”). Since the MWM task is stressful for the mice and we had not seen memory deficits in *Gde2*KO mice at the 7-month time point in the Y maze, we tested the mice only at the 16-month time point (Additional file 1: Fig. S1C). The mice were trained over the course of two days to locate the platform in quadrant one (Fig. 5A; see “Methods”). During training trials, *Gde2*KO mice showed no differences in distance traveled or latency to reach the platform (Fig. 5B; Additional file 1: Fig. S8A), and there was no effect of sex on performance (Additional file 1: Fig. S8B, C). These results suggest that *Gde2*KO mice are capable of learning the platform location.

We next tested *Gde2*KO mice for their ability to remember the platform location after platform removal. During the short delay probe trial at the end of day 2 (see scheme in Fig. 5A), we assessed short-term spatial memory by analyzing the percent time that mice spent in each quadrant area. Both genotypes prefer the correct platform location (quadrant area 1) at higher than chance level; however, only WT mice could accurately distinguish between the first and second quadrants (Fig. 5C). The inability of *Gde2*KOs to discern the first and second quadrants suggests that they have decreased short-term recall of the platform location. This effect is mainly seen in males rather than females (Fig. 5D, E).

We assessed long-term spatial memory by measuring how well mice remembered the platform location after an overnight delay (~ 24 h). Using data collected during the long delay probe trial at the start of day 3 (see scheme in Fig. 5A), we found that similar to the short delay probe trials, WT mice spent significantly more time in the correct quadrant area (quadrant area 1) than the neighboring quadrant area 2 (Fig. 5F). In contrast, *Gde2*KO mice did not show a preference between these two quadrants (Fig. 5F). Thus, *Gde2*KO mice have less precise long-term recall of the platform location than WT mice. This pattern is significant only in males and not females (Fig. 5G, H).

We then measured the ability of animals to learn a new platform location and decrease their preference for the old location of the platform by moving the platform to quadrant 3 on the third day of the MWM test (see scheme in Fig. 5A). Over the course of days 3 and 4, both genotypes learned the new location of the platform (Additional file 1: Fig. S8D) and spent less time around the quadrant area that contained the previous platform location (Additional file 1: Fig. S8G). No sex differences were observed in this reversal portion of the task (Additional file 1: Fig. S8E, F, H, I).

These collective observations show that *Gde2*KO mice, particularly males, fail to recall the specific location of the platform in short- and long-term memory probe trials, while still preserving a general memory of the platform’s location. However, their ability to successfully learn new information is not affected. These findings provide evidence that *Gde2*KO mice have short and long-term spatial memory impairments.

### Loss of GDE2 affects cued fear memory and secondary contextual fear acquisition

**Fig. 6:**
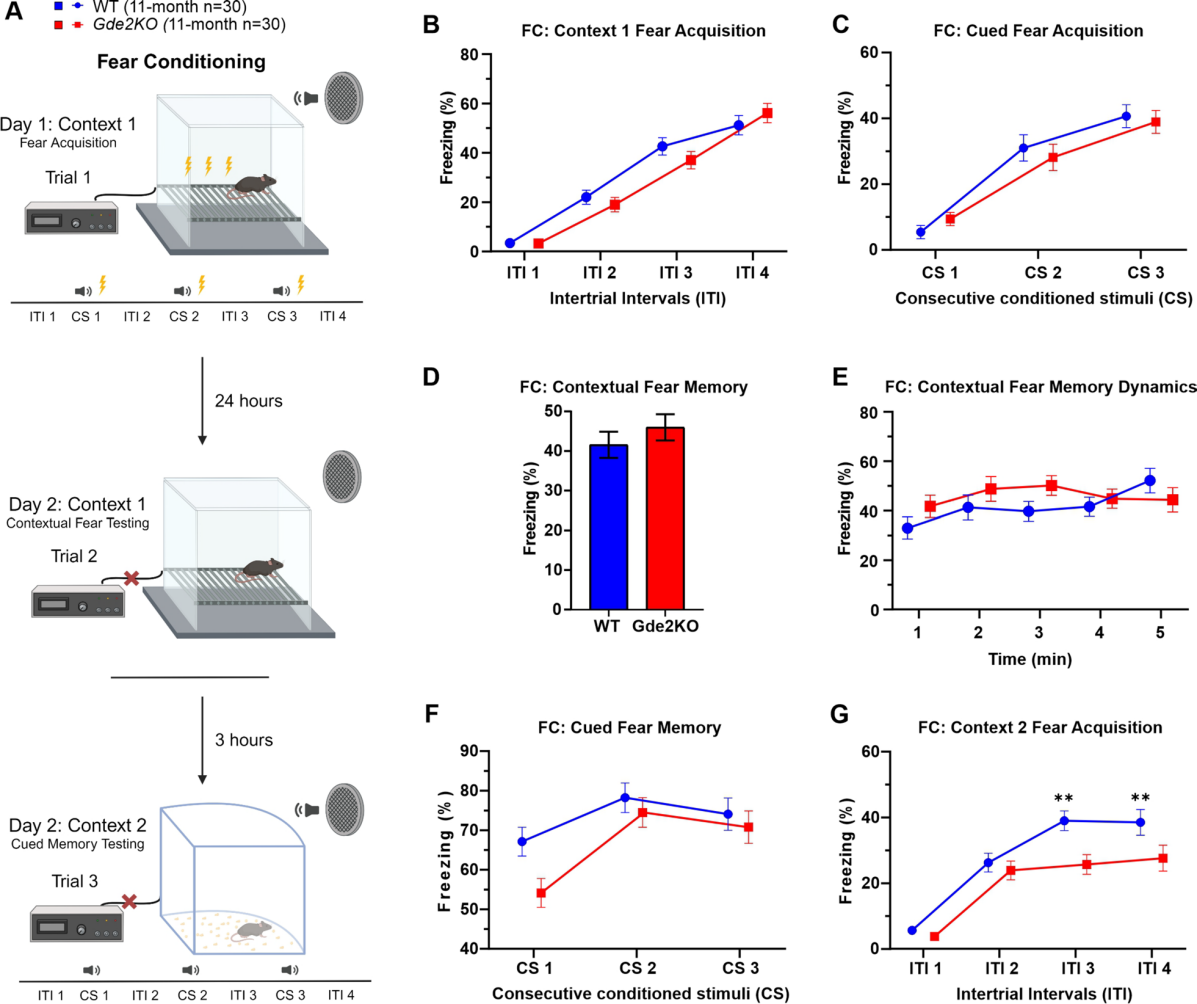
Immediate cued fear memory and secondary contextual fear acquisition deficits in *Gde2*KO mice. **A** Schematic of the FC paradigm. Mice are placed in Context 1 on day 1 and at the start of day 2. In trial 1 (fear acquisition), a tone is played before a shock is delivered to the animals 3 separate times during the test. During trial 2 (contextual fear testing), the mice are placed in the same context, but they receive no sound or shock. During trial 3 (Cued memory testing), the mice are placed in a different box, and only the tone is played while no shock is delivered. **B**, **C** Percent time WT and *Gde2*KO mice spent freezing in the ITI (**B**) and during the CS (**C**) throughout the fear acquisition trial. No effect of genotype or its interactions were detected (three-way mixed design ANOVA, Ps > 0.05). **D**, **E** Percent time spent freezing (**D**) and dynamics (**E**) during the contextual fear testing trial. No effect of genotype or its interactions were detected (three-way mixed design ANOVA, Ps > 0.05). **F** Percent time spent freezing during the CS in trial 3. No effect of genotype or its interactions were detected (three-way mixed design ANOVA, Ps > 0.05). **G** Quantification of time spent freezing during the ITI between tone deliveries during trial 3 revealed a significant decrease in time spent freezing by *Gde2*KO mice as the test progressed (ANOVA, effect of genotype (F(1,56) = 6.16, P < 0.016) and genotype x ITI interaction (F(3,168) = 3.0857, P < 0.0288). All graphs are means ± SEM; ns, *P* > 0.05; and **P < 0.01. See Additional file 2: Table S1 for statistical details. Schematic in **A** created in BioRender.com

GDE2 is expressed in the amygdala (Fig. 1D, E), which is implicated in fear acquisition and memory [34]. Accordingly, we assessed these behaviors using a cued and contextual fear conditioning (FC) test (Fig. 6A, see “Methods”). During the acquisition trial, mice were introduced to context 1 (see scheme in Fig. 6A), where mice received a mild shock (US) after hearing a tone (CS). This was repeated three times throughout the trial, so that mice learned to associate the CS and context with the US. Mice were tested at the 11-month time point to determine if a memory deficit developed after the 7-month time point when no memory deficit was apparent (Additional file 1: Fig. S1C). *Gde2*KO mice of both sexes showed no differences in contextual or cued fear acquisition in context 1 compared with WT controls (Fig. 6B, C; Additional file 1: Fig. S9A–D). We next tested animals for contextual fear memory 24 h after the acquisition trial. In this trial, mice were put back in context 1 but received no CS or US (see scheme in Fig. 6A). We found no significant difference in context 1 fear memory between WT and *Gde2*KO mice of either sex (Fig. 6D, E; Additional file 1: Fig. S9E, F).

We then tested cued fear memory by placing mice in context 2 and playing the CS without any shock (see scheme in Fig. 6A). *Gde2*KO mice, on average, had no significant difference in cued fear memory (Fig. 6F). However, male *Gde2*KO mice froze significantly less during the first CS (Additional file 1: Fig. S9G) indicating that the immediate recall of CS is likely impaired. Females *Gde2*KOs show this trend, but it is not significantly different from WT after Bonferroni correction (Additional file 1: Fig. S9H), which may reflect some sex-specific delay in fear recall in response to the CS. We also assessed freezing during the intertrial interval (ITI). *Gde2*KO mice of both sexes displayed less freezing as the testing progressed compared to their WT counterparts (Fig. 6G, Additional file 1: Fig. S9I, J), consistent with reduced acquisition of secondary contextual fear to context 2.

Overall, *Gde2*KOs showed no difference in the acquisition of cued fear memory or context 1 fear memory. However, when tested for cued fear memory, *Gde2*KOs, particularly males, showed impaired immediate recall of cued fear memory. Moreover, the reduced acquisition of fear response in context 2 might reflect impaired secondary contextual fear acquisition in *Gde2*KO mice.

## Discussion

Previous studies have shown that GDE2 is expressed throughout the developing and early postnatal nervous system [20, 31, 32]. In this study, we find that GDE2 is widely distributed throughout the adult brain, with pronounced expression in the deep layers of the cortex, the dentate gyrus and CA regions of the hippocampus, the thalamus, the habenula, and the amygdala. These brain regions are associated with diverse behaviors. For example, hippocampal areas are implicated in spatial memory, fear memory, and social motivation [35, 36]; the medial habenula and anterior amygdala are involved in fear/anxiety and sociability [33, 34], the cortex is associated with multiple aspects of information processing [44–46], while the thalamus is involved in the startle reflex and sensorimotor gating [47, 48]. Impairments in many of these brain regions can result in behavioral changes associated with disease. Further, GDE2 is aberrantly mis-localized in AD, ALS, and ALS/FTD post-mortem brain and appears dysfunctional in these diseases [21, 22]. Accordingly, these collective observations motivated us to investigate the consequences of GDE2 ablation in a range of behavioral tests, some of which evaluate behaviors known to be affected in disease.

We find that *Gde2*KO mice of both sexes exhibit a hyperactive phenotype that can be observed across multiple tests including OF, plus maze, and Y maze. Older *Gde2*KO mice demonstrate a more robust hyperactive phenotype while younger mice tend to show increased hyperactivity mainly in novel environments. *Gde2*KO mice also show abnormal startle response, and females show PPI deficits suggestive of possible sensorimotor gating deficits. Both male and female *Gde2*KO mice show a decrease in sociability as measured in the social motivation task. Further, *Gde2*KO animals show deficits in long- and short-term spatial working memory at 16 months of age. In the 16-month Y maze task, WT mice spend more time in the novel arm throughout the second trial while *Gde2*KO mice only differentiate between the novel and old arm at the start of the trial. Additionally, in the MWM, male *Gde2*KO mice are less accurate at finding the correct quadrant area than WT mice. Finally, we found that *Gde2*KO mice exhibit a deficit in the immediate recall of cued fear response and the acquisition of secondary contextual fear, which is demonstrated by freezing in response to the CS being delivered in a novel context. Altogether, our results show that adult *Gde2*KO mice display a variety of behavioral and memory deficits that could arise from the dysfunction of specific brain regions where GDE2 is normally expressed.

In general, correlating the loss of a protein in a particular brain area with a specific behavioral phenotype is difficult. However, this study supports a role for GDE2 in contributing to multiple behavioral phenotypes, some of which are associated with neurodegenerative disease pathologies. Notably, several of the phenotypes observed are found in neurodegenerative mouse models. For example, the AD model *5XFAD* shows hyperactivity, startle and PPI deficits, reduced sociability, and impaired spatial working memory [9, 49–52]. The *APP/PS1* AD model also shows hyperactivity, reduced PPI, and spatial memory deficits [9–11, 53]. Further, the ALS/FTD model *TDP-43-ΔNLS* and tauopathy model *P301S* show hyperactivity, decreased sociability, and impaired spatial memory [54–58]. These overlapping phenotypes are of interest as GDE2 distribution and function are impaired in AD, ALS, and ALS/FTD patients [21, 22]. Interestingly the behavioral phenotypes observed in *Gde2*KO mice were detected without additional co-expression of any other proteins that are associated with causative players in AD, ALS, and ALS/FTD models. This suggests that GDE2 disruption could have additive phenotypic effects on behavior and cognition in a disease context. One limitation to the current study is that the WT and *Gde2*KO mice tested were not littermates. Since we needed ~ 70 animals all born within a short time period to ensure appropriate cohort sizes, it was not feasible to rely on heterozygous crosses to generate this number of animals. All mice had the same genetic background and were generated from the same number of crosses. Therefore, it is likely this limitation will not confound the results of our study.

Building upon this work, future studies will aim to conduct behavioral tests on temporal and cell-type specific *Gde2*KOs to differentiate between developmental versus post-developmental and cell-type specific contributions of GDE2 to behavioral and cognitive function. Additionally, further investigation of the cellular and molecular changes related to loss of GDE2 would be informative. Synaptic function deficits are commonly found in neurodegenerative diseases and are known to contribute to behavioral and cognitive defects [59, 60]. *Gde2*KO animals show alterations in synaptic proteins and synapse numbers [21]. Accordingly, a deeper investigation of the role GDE2 plays in synaptic physiology is needed, in concert with the continued study of GDE2 in the initiation and progression of neurodegenerative disease.

## Conclusions

We found that GDE2 is widely expressed throughout the adult mouse brain including in regions implicated in controlling complex behaviors and cognition. *Gde2*KO mice exhibited hyperactivity, decreased PPI and startle response, reduced sociability, impaired spatial memory, and deficits in cued fear memory/secondary contextual fear acquisition. Many of the behavioral phenotypes observed in *Gde2*KO mice are also found in models of neurodegenerative diseases, specifically models of AD and ALS/FTD. Together with previous work suggesting GDE2 dysfunction in AD and ALS/FTD patients, our observations provide behavioral evidence supporting GDE2 contributions to neurodegenerative phenotypes associated with disease.

### Supplementary Information

**Additional file 1: Figure S1.** Reduced body weight in male *Gde2*KO mice. **Figure S2.** Sex differences in *Gde2*KO hyperactivity phenotype. **Figure S3.** Sex differences in *Gde2*KO plus maze hyperactivity phenotype and condensed dynamic. **Figure S4.** Anxiety metrics in OF and plus maze. **Figure S5.** Differences in PPI are not explained by differences in startle response for *Gde2*KO animals. **Figure S6.** Sex separation and dynamics for social motivation measurements. **Figure S7.** Sex-separated analysis of Y Maze spatial preferences. **Figure S8.** *Gde2*KO mice learn new platform location during reversal days. **Figure S9.** Sex-separated analysis of FC task.

**Additional file 2: Table S1. **Statistics for main and additional figures.

## Data Availability

The datasets used and/or analyzed in this study are available from the corresponding author upon reasonable request.

## Abbreviations

AD: Alzheimer’s disease
ADAM10: A Disintegrin and metalloprotease domain-containing protein 10
ALS: Amyotrophic lateral sclerosis
ALS/FTD: Amyotrophic Lateral Sclerosis/Frontotemporal Dementia
APP: Amyloid precursor protein
ASR: Acoustic startle reaction
CP: Caudate putamen
CS: Conditioned stimulus
CSF: Cerebrospinal fluid
FC: Fear conditioning
GDE2: Glycerophosphodiester phosphodiesterase 2
GPI: Glycosylphosphatidylinositol
hGDE2: Human GDE2
ITI: Intertrial interval
MAPT: Microtubule associated protein tau
MWM: Morris Water Maze
OF: Open Field
PB: Phosphate buffer
PFA: Paraformaldehyde
PPI: Pre-pulse inhibition
PSEN1: Presenelin1
RECK: Reversion inducing cysteine rich protein with Kazal motifs
SOD1: Superoxide dismutase1
Th: Thalamus
US: Unconditioned stimulus

## References

[1] Knopman DS, Amieva H, Petersen RC, Chételat G, Holtzman DM, Hyman BT (2021). Alzheimer disease. Nat Rev Dis Primers.

[2] Taylor JP, Brown RH, Cleveland DW (2016). Decoding ALS: from genes to mechanism. Nature.

[3] Sivasathiaseelan H, Marshall CR, Agustus JL, Benhamou E, Bond RL, van Leeuwen JEP (2019). Frontotemporal dementia: a clinical review. Semin Neurol.

[4] Sherrington R, Rogaev E, Liang Y, Rogaeva E, Levesque G, Ikeda M (1995). Cloning of a gene bearing missense mutations in early-onset familial Alzheimer's disease. Nature.

[5] Goate A, Chartier-Harlin M-C, Mullan M, Brown J, Crawford F, Fidani L (1991). Segregation of a missense mutation in the amyloid precursor protein gene with familial Alzheimer's disease. Nature.

[6] Rosen DR, Siddique T, Patterson D, Figlewicz DA, Sapp P, Hentati A (1993). Mutations in Cu/Zn superoxide dismutase gene are associated with familial amyotrophic lateral sclerosis. Nature.

[7] Spillantini MG, Murrell JR, Goedert M, Farlow MR, Klug A, Ghetti B (1998). Mutation in the tau gene in familial multiple system tauopathy with presenile dementia. Proc Natl Acad Sci.

[8] Dawson TM, Golde TE, Lagier-Tourenne C (2018). Animal models of neurodegenerative diseases. Nat Neurosci.

[9] Kosel F, Pelley JMS, Franklin TB (2020). Behavioural and psychological symptoms of dementia in mouse models of Alzheimer's disease-related pathology. Neurosci Biobehav Rev.

[10] Wang T, Chen Y, Zou Y, Pang Y, He X, Liu Y (2022). Locomotor hyperactivity in the early-stage Alzheimer's disease-like pathology of APP/PS1 mice: associated with impaired polarization of astrocyte aquaporin 4. Aging Dis.

[11] Wang H, He J, Zhang R, Zhu S, Wang J, Kong L (2012). Sensorimotor gating and memory deficits in an APP/PS1 double transgenic mouse model of Alzheimer's disease. Behav Brain Res.

[12] Jacobsen JS, Wu C-C, Redwine JM, Comery TA, Arias R, Bowlby M (2006). Early-onset behavioral and synaptic deficits in a mouse model of Alzheimer's disease. Proc Natl Acad Sci.

[13] Webster SJ, Bachstetter AD, Van Eldik LJ (2013). Comprehensive behavioral characterization of an APP/PS-1 double knock-in mouse model of Alzheimer's disease. Alzheimers Res Ther.

[14] Park S, Lee C, Sabharwal P, Zhang M, Meyers CLF, Sockanathan S (2013). GDE2 promotes neurogenesis by glycosylphosphatidylinositol-anchor cleavage of RECK. Science.

[15] Matas-Rico E, van Veen M, Leyton-Puig D, van den Berg J, Koster J, Kedziora KM (2016). Glycerophosphodiesterase GDE2 promotes neuroblastoma differentiation through glypican release and is a marker of clinical outcome. Cancer Cell.

[16] Yanaka N (2007). Mammalian glycerophosphodiester phosphodiesterases. Biosci Biotechnol Biochem.

[17] Rao M, Sockanathan S (2005). Transmembrane protein GDE2 induces motor neuron differentiation in vivo. Science.

[18] Sabharwal P, Lee C, Park S, Rao M, Sockanathan S (2011). GDE2 regulates subtype-specific motor neuron generation through inhibition of Notch signaling. Neuron.

[19] Rodriguez M, Choi J, Park S, Sockanathan S (2012). Gde2 regulates cortical neuronal identity by controlling the timing of cortical progenitor differentiation. Development.

[20] Cave C, Park S, Rodriguez M, Nakamura M, Hoke A, Pletnikov M (2017). GDE2 is essential for neuronal survival in the postnatal mammalian spinal cord. Mol Neurodegener.

[21] Nakamura M, Li Y, Choi B-R, Matas-Rico E, Troncoso J, Takahashi C (2021). GDE2-RECK controls ADAM10 α-secretase-mediated cleavage of amyloid precursor protein. Sci Transl Med..

[22] Westerhaus A, Joseph T, Meyers AJ, Jang Y, Na CH, Cave C (2022). The distribution and function of GDE2, a regulator of spinal motor neuron survival, are disrupted in Amyotrophic Lateral Sclerosis. Acta Neuropathol Commun.

[23] Kazuki Y, Gao FJ, Li Y, Moyer AJ, Devenney B, Hiramatsu K (2020). A non-mosaic transchromosomic mouse model of Down syndrome carrying the long arm of human chromosome 21. Elife.

[24] Singh B, Covelo A, Martell-Martínez H, Nanclares C, Sherman MA, Okematti E (2019). Tau is required for progressive synaptic and memory deficits in a transgenic mouse model of α-synucleinopathy. Acta Neuropathol.

[25] Savonenko AV, Xu GM, Price DL, Borchelt DR, Markowska AL (2003). Normal cognitive behavior in two distinct congenic lines of transgenic mice hyperexpressing mutant APPSWE. Neurobiol Dis.

[26] Moy SS, Nadler JJ, Perez A, Barbaro RP, Johns JM, Magnuson TR (2004). Sociability and preference for social novelty in five inbred strains: an approach to assess autistic-like behavior in mice. Genes Brain Behav.

[27] Savonenko A, Xu GM, Melnikova T, Morton JL, Gonzales V, Wong MP (2005). Episodic-like memory deficits in the APPswe/PS1dE9 mouse model of Alzheimer's disease: relationships to beta-amyloid deposition and neurotransmitter abnormalities. Neurobiol Dis.

[28] Brose RD, Savonenko A, Devenney B, Smith KD, Reeves RH (2019). Hydroxyurea improves spatial memory and cognitive plasticity in mice and has a mild effect on these parameters in a Down syndrome mouse model. Front Aging Neurosci.

[29] Gao FJ, Klinedinst D, Fernandez FX, Cheng B, Savonenko A, Devenney B (2021). Forebrain Shh overexpression improves cognitive function and locomotor hyperactivity in an aneuploid mouse model of Down syndrome and its euploid littermates. Acta Neuropathol Commun.

[30] Zhang W, Chuang YA, Na Y, Ye Z, Yang L, Lin R (2019). Arc oligomerization is regulated by CaMKII phosphorylation of the GAG domain: an essential mechanism for plasticity and memory formation. Mol Cell.

[31] Choi BR, Dobrowolski M, Sockanathan S (2021). GDE2 expression in oligodendroglia regulates the pace of oligodendrocyte maturation. Dev Dyn.

[32] Choi B-R, Cave C, Na CH, Sockanathan S (2020). GDE2-dependent activation of canonical wnt signaling in neurons regulates oligodendrocyte maturation. Cell Rep.

[33] Ables JL, Park K, Ibañez-Tallon I (2023). Understanding the habenula: a major node in circuits regulating emotion and motivation. Pharmacol Res.

[34] Janak PH, Tye KM (2015). From circuits to behaviour in the amygdala. Nature.

[35] Knierim JJ (2015). The hippocampus. Curr Biol.

[36] Montagrin A, Saiote C, Schiller D (2018). The social hippocampus. Hippocampus.

[37] Yao Z, van Velthoven CTJ, Nguyen TN, Goldy J, Sedeno-Cortes AE, Baftizadeh F (2021). A taxonomy of transcriptomic cell types across the isocortex and hippocampal formation. Cell.

[38] Ye Z, Kenian C, Steven AS, Mariko LB, Anja RS, Sean O (2014). An RNA-sequencing transcriptome and splicing database of glia, neurons, and vascular cells of the cerebral cortex. J Neurosci.

[39] Prut L, Belzung C (2003). The open field as a paradigm to measure the effects of drugs on anxiety-like behaviors: a review. Eur J Pharmacol.

[40] Kraeuter AK, Guest PC, Sarnyai Z (2019). The elevated plus maze test for measuring anxiety-like behavior in rodents. Methods Mol Biol.

[41] Geyer MA, Krebs-Thomson K, Braff DL, Swerdlow NR (2001). Pharmacological studies of prepulse inhibition models of sensorimotor gating deficits in schizophrenia: a decade in review. Psychopharmacology.

[42] Morris R (1984). Developments of a water-maze procedure for studying spatial learning in the rat. J Neurosci Methods.

[43] Frick KM, Baxter MG, Markowska AL, Olton DS, Price DL (1995). Age-related spatial reference and working memory deficits assessed in the water maze. Neurobiol Aging.

[44] Miller EK (2000). The prefrontal cortex and cognitive control. Nat Rev Neurosci.

[45] Miller EK, Cohen JD (2001). An integrative theory of prefrontal cortex function. Annu Rev Neurosci.

[46] Fuster J (2015). The prefrontal cortex.

[47] Hazlett EA, Buchsbaum MS, Tang CY, Fleischman MB, Wei T-C, Byne W (2001). Thalamic activation during an attention-to-prepulse startle modification paradigm: a functional MRI study. Biol Psychiat.

[48] You Q-L, Luo Z-C, Luo Z-Y, Kong Y, Li Z-L, Yang J-M (2021). Involvement of the thalamic reticular nucleus in prepulse inhibition of acoustic startle. Transl Psychiatry..

[49] Flanigan TJ, Xue Y, Kishan Rao S, Dhanushkodi A, McDonald MP (2014). Abnormal vibrissa-related behavior and loss of barrel field inhibitory neurons in 5xFAD transgenics. Genes Brain Behav.

[50] O'Leary TP, Shin S, Fertan E, Dingle RN, Almuklass A, Gunn RK (2017). Reduced acoustic startle response and peripheral hearing loss in the 5xFAD mouse model of Alzheimer's disease. Genes Brain Behav.

[51] Kosel F, Torres Munoz P, Yang JR, Wong AA, Franklin TB (2019). Age-related changes in social behaviours in the 5xFAD mouse model of Alzheimer’s disease. Behav Brain Res.

[52] Oakley H, Cole SL, Logan S, Maus E, Shao P, Craft J (2006). Intraneuronal beta-amyloid aggregates, neurodegeneration, and neuron loss in transgenic mice with five familial Alzheimer's disease mutations: potential factors in amyloid plaque formation. J Neurosci.

[53] Huang H, Nie S, Cao M, Marshall C, Gao J, Xiao N (2016). Characterization of AD-like phenotype in aged APPSwe/PS1dE9 mice. Age (Dordr).

[54] Alfieri JA, Pino NS, Igaz LM (2014). Reversible behavioral phenotypes in a conditional mouse model of TDP-43 proteinopathies. J Neurosci.

[55] Przybyla M, Stevens CH, van der Hoven J, Harasta A, Bi M, Ittner A (2016). Disinhibition-like behavior in a P301S mutant tau transgenic mouse model of frontotemporal dementia. Neurosci Lett.

[56] Watt G, Przybyla M, Zak V, van Eersel J, Ittner A, Ittner LM (2020). Novel behavioural characteristics of male human P301S mutant Tau transgenic mice—a model for tauopathy. Neuroscience.

[57] Schindowski K, Bretteville A, Leroy K, Bégard S, Brion J-P, Hamdane M (2006). Alzheimer's disease-like tau neuropathology leads to memory deficits and loss of functional synapses in a novel mutated tau transgenic mouse without any motor deficits. Am J Pathol.

[58] Cook C, Dunmore JH, Murray ME, Scheffel K, Shukoor N, Tong J (2014). Severe amygdala dysfunction in a MAPT transgenic mouse model of frontotemporal dementia. Neurobiol Aging.

[59] Chapman PF, White GL, Jones MW, Cooper-Blacketer D, Marshall VJ, Irizarry M (1999). Impaired synaptic plasticity and learning in aged amyloid precursor protein transgenic mice. Nat Neurosci.

[60] Lerdkrai C, Asavapanumas N, Brawek B, Kovalchuk Y, Mojtahedi N, Olmedillas Del Moral M (2018). Intracellular Ca(2+) stores control in vivo neuronal hyperactivity in a mouse model of Alzheimer's disease. Proc Natl Acad Sci USA.

