# Supplementary material for: Loss of GDE2 leads to complex behavioral changes including memory impairment
Source: Behav Brain Funct. 2024 Apr 4;20:7. doi: 10.1186/s12993-024-00234-1 (PMC10993612; doi:10.1186/s12993-024-00234-1)
Supplement: Supplementary file 1 — Additional file 1: Figure S1. Reduced body weight in male Gde2KO mice. Figure S2. Sex differences in Gde2KO hyperactivity phenotype. Figure S3. Sex differences in Gde2KO plus maze hyperactivity phenotype and condensed dynamic. Figure S4. Anxiety metrics in OF and plus maze. Figure S5. Differences in PPI are not explained by differences in startle response for Gde2KO animals. Figure S6. Sex separation and dynamics for social motivation measurements. Figure S7. Sex-separated analysis of Y Maze spatial preferences. Figure S8. Gde2KO mice learn new platform location during reversal days. Figure S9. Sex-separated analysis of FC task. [file 12993_2024_234_MOESM1_ESM.pdf]

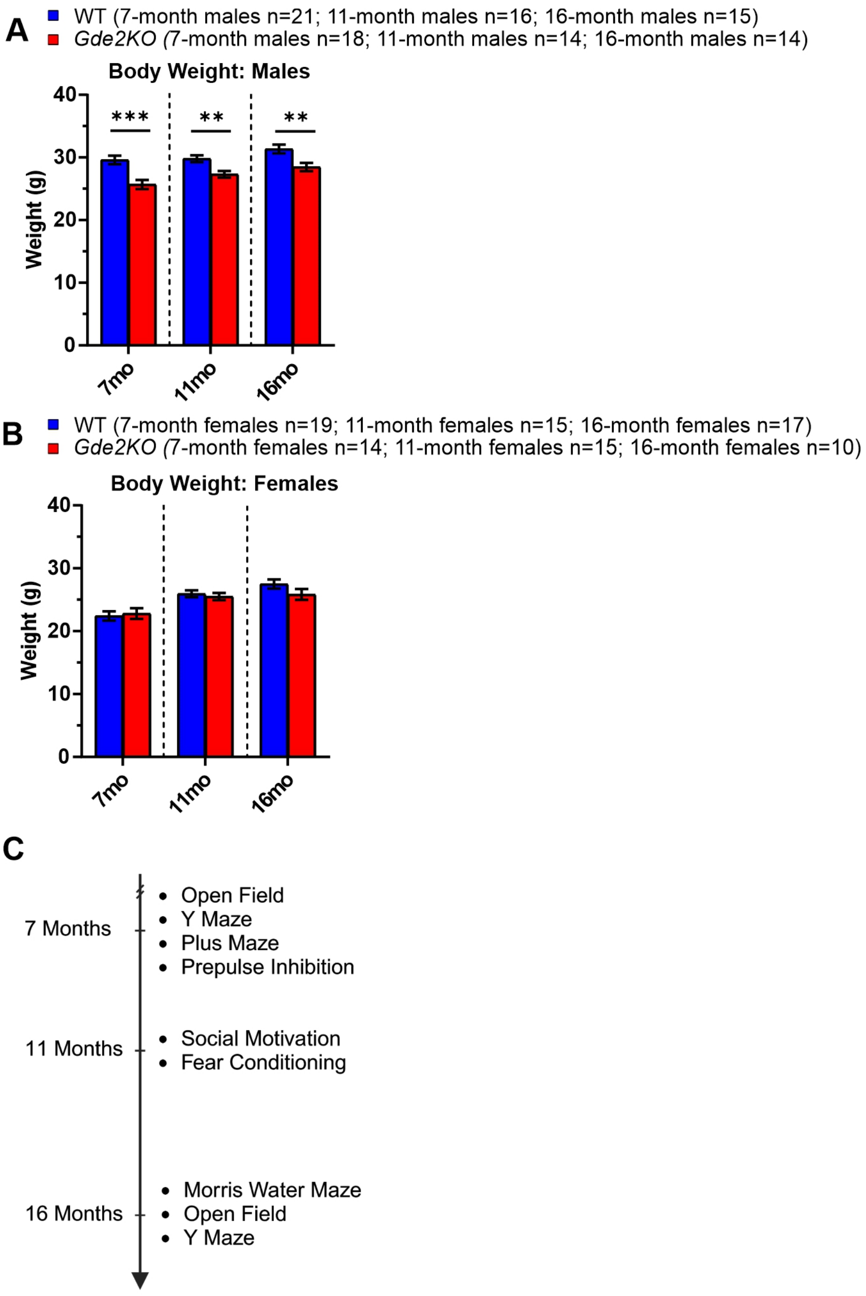

Fig. S1: Reduced body weight in male *Gde2*KO mice

(A-B) Body weight of WT and *Gde2*KO mice measured at 7, 11, and 16 months separated by males (A) and females (B) respectively. Male WT mice weigh significantly more than *Gde2*KO mice at all ages (Ps<0.01 Fisher post hoc). (C) Timeline of when each behavioral task was

performed. All graphs are means ± SEM; ns, $P > 0.05$; *$P < 0.05$, **$P < 0.01$, and ***$P < 0.001$.

See Table S1 for statistical details.

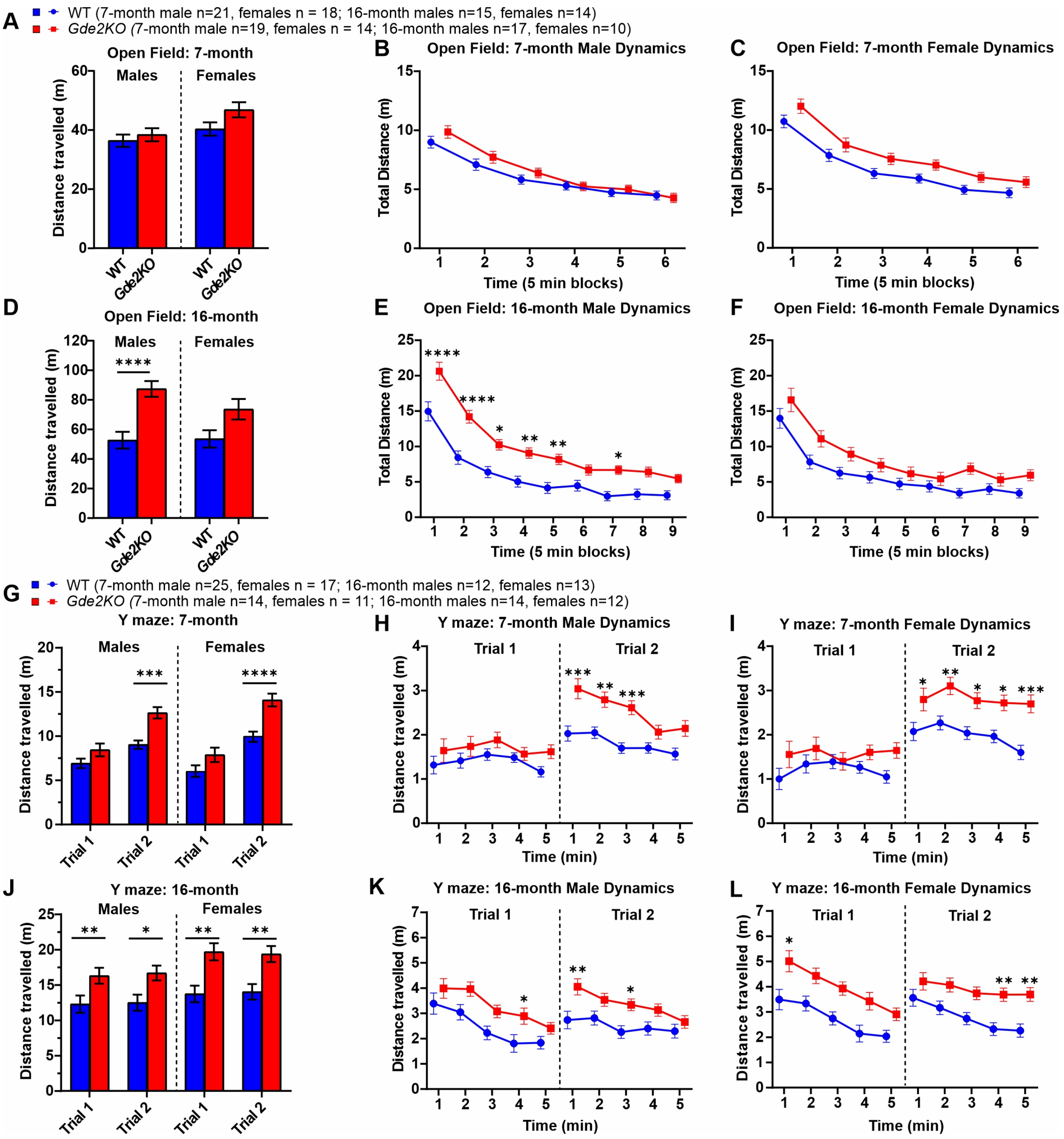

Fig. S2: Sex differences in *Gde2*KO hyperactivity phenotype

(A-F) Analysis of distance traveled in OF test separated by sex. (A-C) Total distance (A) and dynamics for males (B) and females (C) during 30 minutes of testing in the OF task for 7-month-old WT and *Gde2*KO mice. No effect of genotype or its interactions was detected (three-way mixed design ANOVA, Ps>0.05). (D-F) The same measures and analysis as in A-C for 16-

month-old mice. *Gde2*KO male mice demonstrated higher motor activity (D, Fisher LSD post hoc P<0.0001) that was partially ameliorated as testing progressed (E). While *Gde2*KO female mice (D, F) showed a trend towards higher motor activity (Fisher LSD post hoc, P<0.033 pre-Bonferonni correction), this was not significant after Bonferroni correction (P > 0.05). (G-L) Analysis of distance traveled in Y maze task separated by sex. (G-I) Total distance (G) and dynamics for males (H) and females (I) during Y maze testing in 7-month-old mice. *Gde2*KO male and female mice were more active than WT mice during trial 2 (ANOVA, effect of genotype $F(1,\geq 24)>18.18$, P<0.0002) but not during trial 1 (Ps >0.05). (J-L) The same measures and analysis as in G-I for 16-month-old mice. *Gde2*KO male and female mice were more active than WT mice during both trial 1 (ANOVA, effect of genotype $F(1,\geq 23)>8.96$, P<0.007) and trial 2 (ANOVA, effect of genotype $F(1,\geq 23)>6.66$, P<0.02). All graphs are means ± SEM; ns, $P > 0.05$; *$P < 0.05$, **$P < 0.01$, ***$P < 0.001$, and ****$P < 0.0001$. See Table S1 for statistical details.

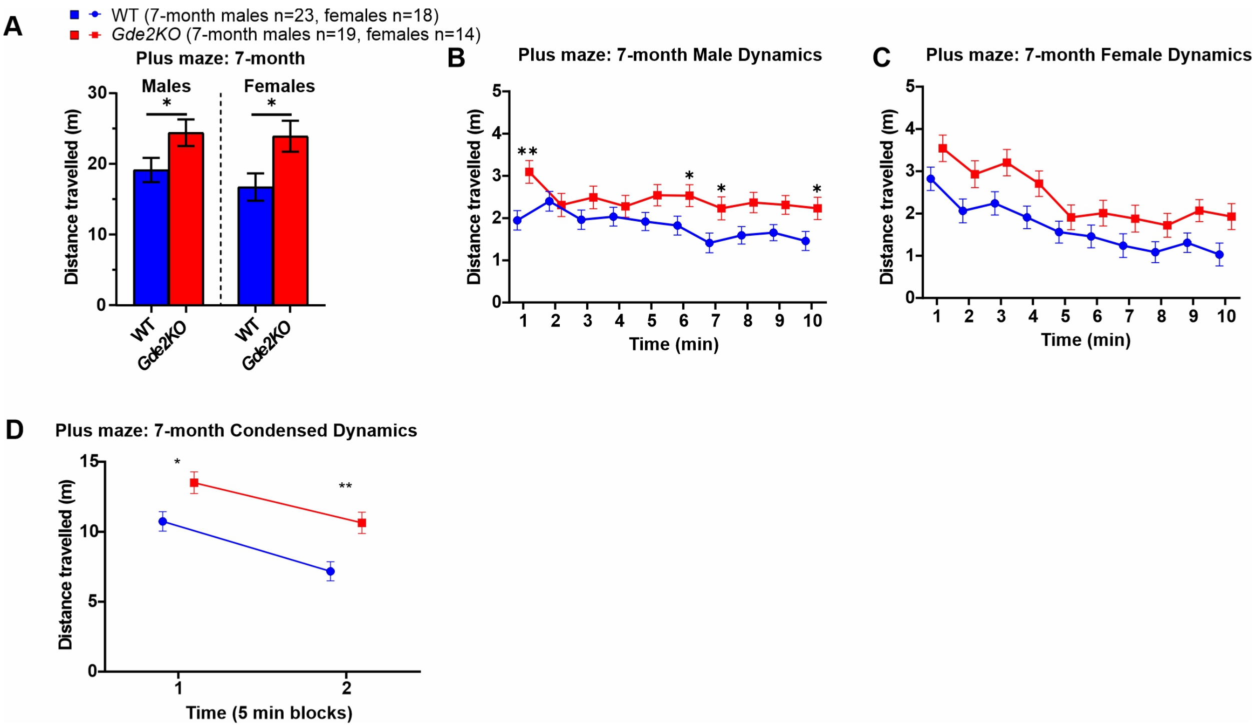

Fig. S3: Sex differences in *Gde2*KO plus maze hyperactivity phenotype and condensed dynamic

(A-C) Analysis of distance traveled in plus maze task separated by sex. Total distance (A) and dynamics for males (B) and females (C) during 10 minutes of testing in the plus maze in 7-month-old mice. *Gde2*KO male and female mice were both more active than WT mice (ANOVA, effect of genotype $F(1,\geq30)>4.25$, $P<0.05$). (D) Condensed dynamics of distance traveled by mice in plus maze. All graphs are means ± SEM; ns, $P > 0.05$; *$P < 0.05$, and **$P < 0.01$. See Table S1 for statistical details.

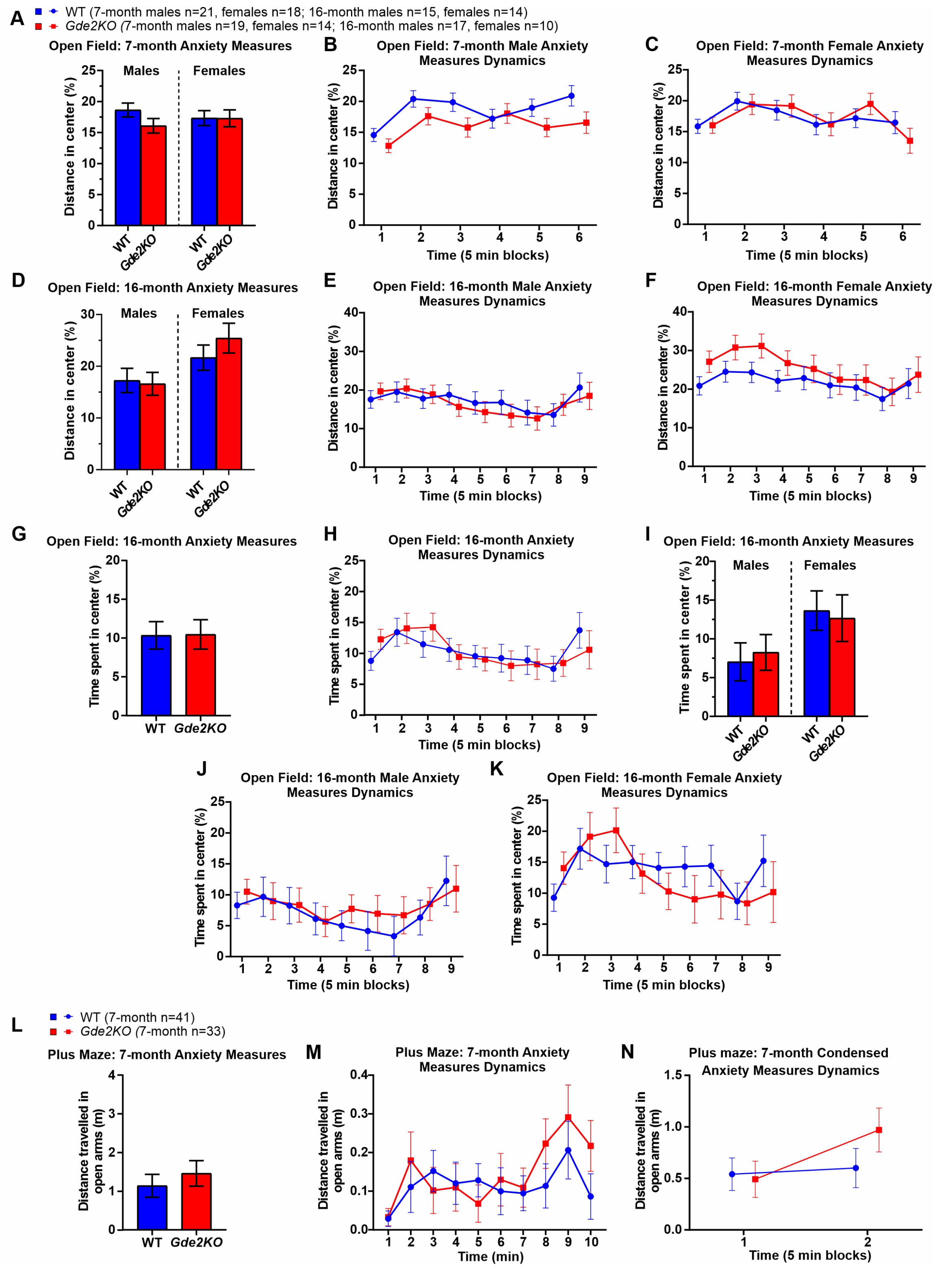

Fig. S4: Anxiety metrics in OF and plus maze

(A-K) Analysis of anxiety phenotype in OF test separated by sex. (A-C) Total percent distance traveled in the center of the OF setup (A) and its dynamics for males (B) and females (C) at 7 months. No effect of genotype or its interactions was detected (three-way mixed design ANOVA, $P>0.05$). (D-F) The same measurements and analysis as in A-C, respectively, for 16-month-old mice (also ns). (G-K) Total time spent in the center of OF setup (G) and its dynamics (H) at 16 months. No effect of genotype or its interactions was detected (ANOVA, $P>0.05$). Same measurements and analysis as in G and H for the total time spent in the center separated by sex (I) and its dynamics for males (J) and females (K) (also ns). (L-N) Total distance traveled (L), dynamics (M), and condensed dynamics (N) in the open arms of the plus maze. No effect of genotype or its interactions was detected (ANOVA, $P>0.05$). All graphs are means ± SEM; ns, $P > 0.05$; *$P < 0.05$, **$P < 0.01$, ***$P < 0.001$, and ****$P < 0.0001$. See Table S1 for statistical details.

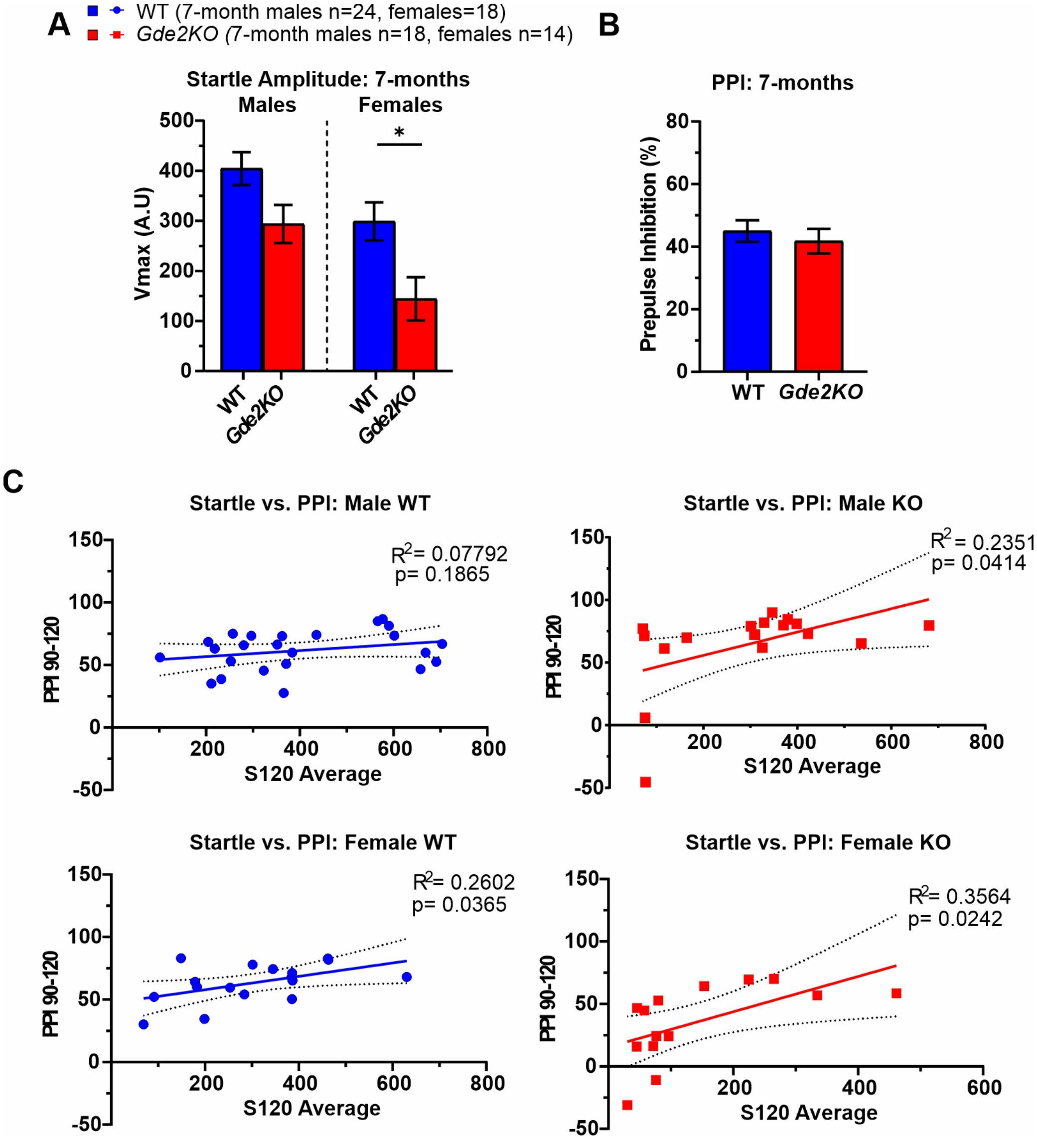

Fig. S5: Differences in PPI are not explained by differences in startle response for *Gde2*KO animals

(A) Average startle response in mice when the 120 dB pulse stimulus was delivered with no prepulse, separated by sex. Female *Gde2*KO mice show a decrease in startle amplitude (Fisher LSD post hoc, P<0.019), while males show a trending decrease (Fisher LSD post hoc pre-Bonferroni correction, P<0.032, P>0.05 after correction). (B) Analysis of PPI with sexes combined in WT and *Gde2*KO mice. No effect of genotype was detected (ANOVA, P>0.05) (C)

Linear regressions of Startle vs. PPI separated by genotype and sex. All graphs are means ± SEM; ns, $P > 0.05$; *$P < 0.05$, **$P < 0.01$, and ***$P < 0.001$. See Table S1 for statistical details.

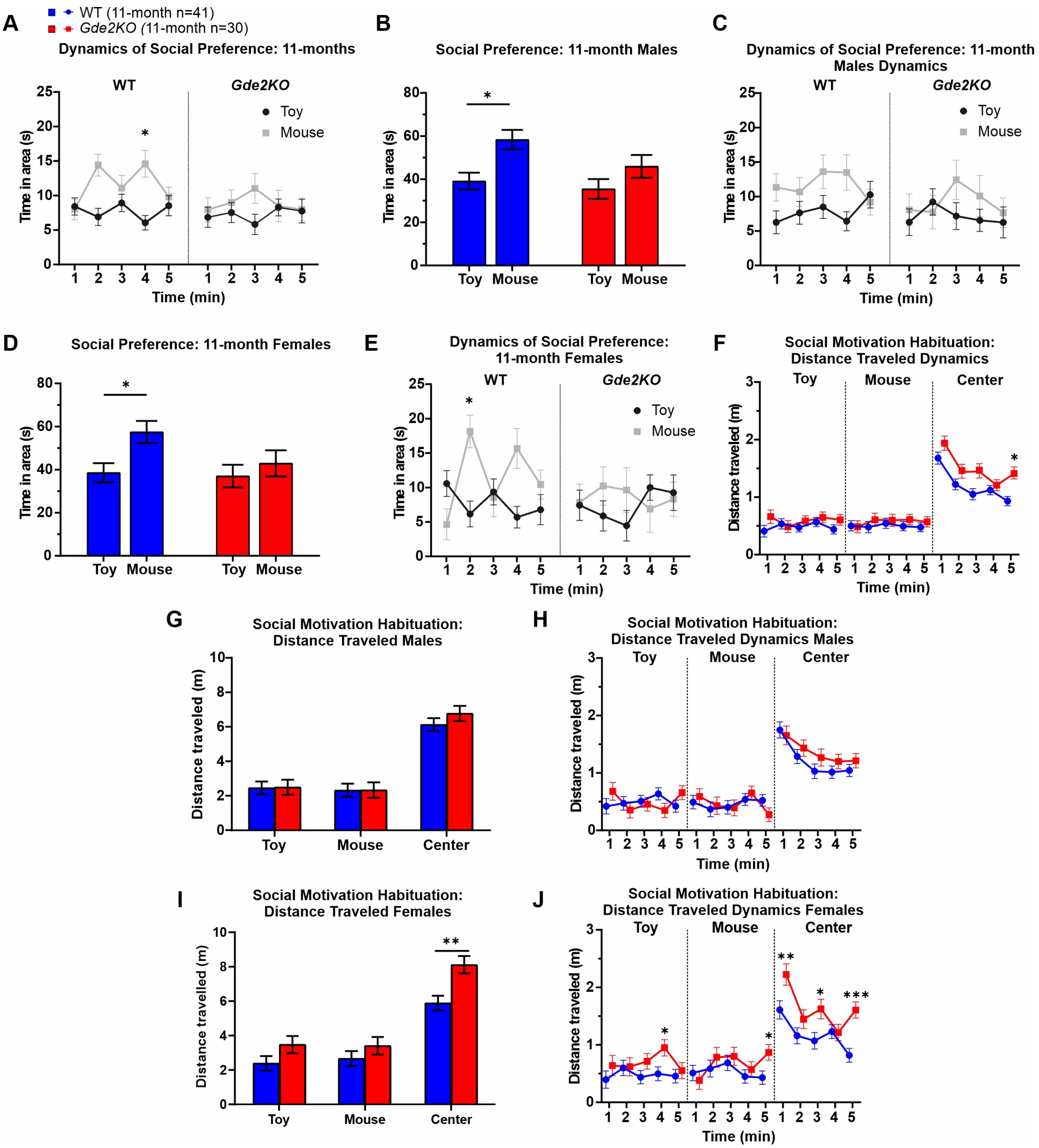

Fig. S6: Sex separation and dynamics for social motivation measurements

(A-E) Time spent by WT and *Gde2*KO mice in the area directly around the toy and mouse chambers during the social motivation trial. Dynamics of time spent in toy and mouse areas where sex has been combined (A) and separated by males (C) and females (E). Total time

spent in toy and mouse area for males (B) and females (D). Both male and female WT mice show an increase in time spent in the mouse area compared to the toy area (Fisher LSD post hoc, $P<0.03$), while *Gde2*KO males and females showed no difference in time spent between the two areas ($Ps>0.05$). (F-J) Distance traveled by mice in each compartment during the habituation trial. Dynamics of distance traveled by mice in all compartments where sex has been combined (F) and separated by males (H) and females (J). Analysis of total distance traveled in the three chambers separated by males (G) and females (I). Female *Gde2*KO mice show increased distance traveled in the center compartment (Fisher LSD post hoc, $P<0.006$). All graphs are means ± SEM; ns, $P > 0.05$; *$P < 0.05$, **$P < 0.01$, and ***$P < 0.001$. See Table S1 for statistical details.

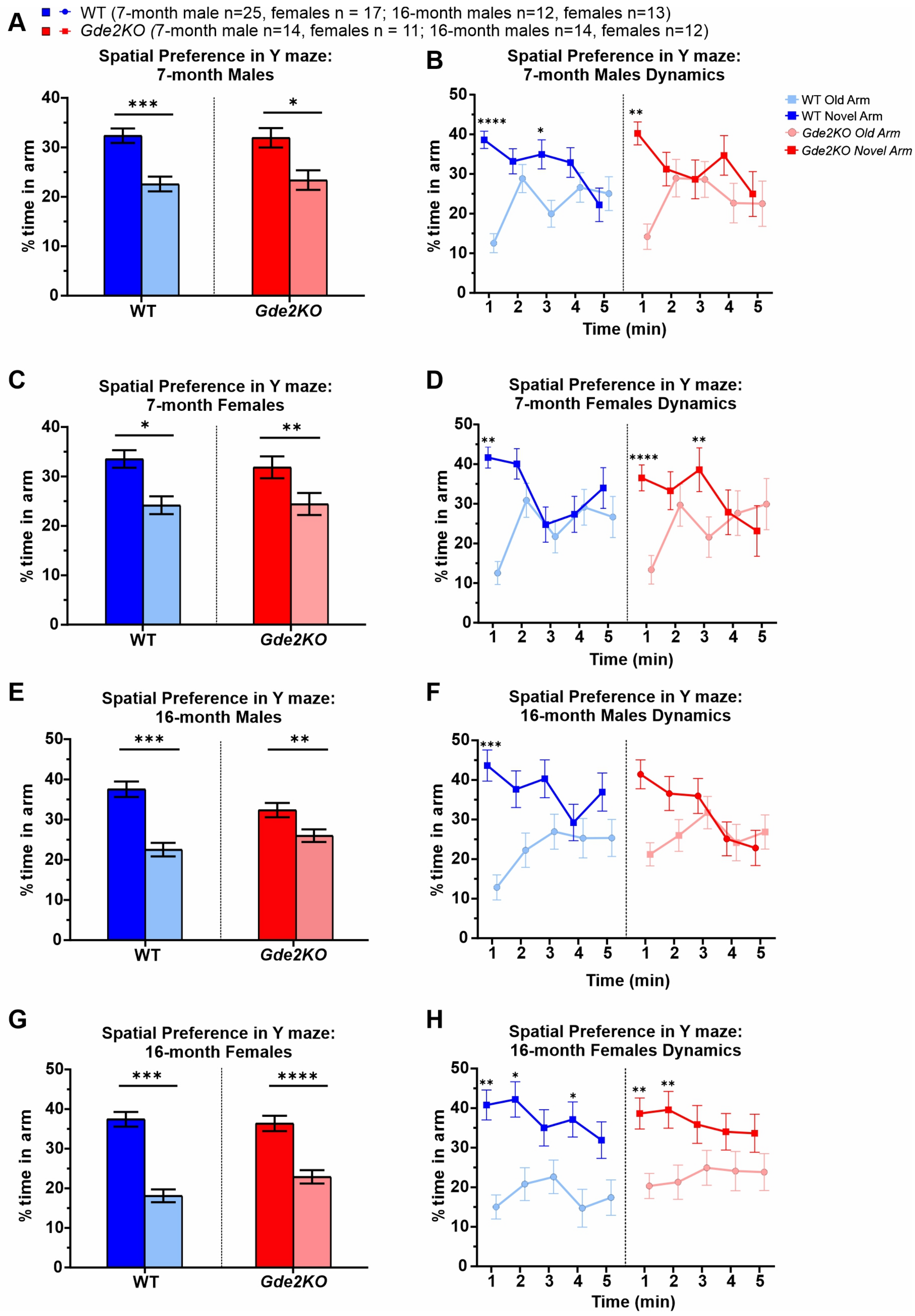

A
WT (7-month male n=25, females n = 17; 16-month males n=12, females n=13)
Gde2KO (7-month male n=14, females n = 11; 16-month males n=14, females n=12)
Spatial Preference in Y maze: 7-month Males
Spatial Preference in Y maze: 7-month Males Dynamics
Spatial Preference in Y maze: 7-month Females
Spatial Preference in Y maze: 7-month Females Dynamics
Spatial Preference in Y maze: 16-month Males
Spatial Preference in Y maze: 16-month Males Dynamics
Spatial Preference in Y maze: 16-month Females
Spatial Preference in Y maze: 16-month Females Dynamics
WT Old Arm
WT Novel Arm
Gde2KO Old Arm
Gde2KO Novel Arm
% time in arm
Time (min)
WT
Gde2KO
B
C
D
E
F
G
H

Fig. S7: Sex-separated analysis of Y Maze spatial preferences

(A-D) Spatial preference of WT and *Gde2*KO mice as measured by percent time spent in the novel and old arms at the 7-month time point. Total percent time spent in each arm separated by males (A) and females (C), and dynamics for males (B) and females (D). WT and *Gde2*KO mice of both sexes spent significantly more time in the novel arm compared to the old arm (ANOVA, effect of arm, $F(1,\geq 10)>5.69$, $P<0.03$) (E-H) The same measurements and analysis as in A-D, respectively, for 16-month-old mice. WT and *Gde2*KO mice of both sexes spent significantly more time in the novel arm compared to the old arm (ANOVA, effect of arm, $F(1,\geq 11)>12.5$, $P<0.004$). All graphs are means ± SEM; ns, $P > 0.05$; *$P < 0.05$, **$P < 0.01$, ***$P < 0.001$, and ****$P < 0.0001$. See Table S1 for statistical details.

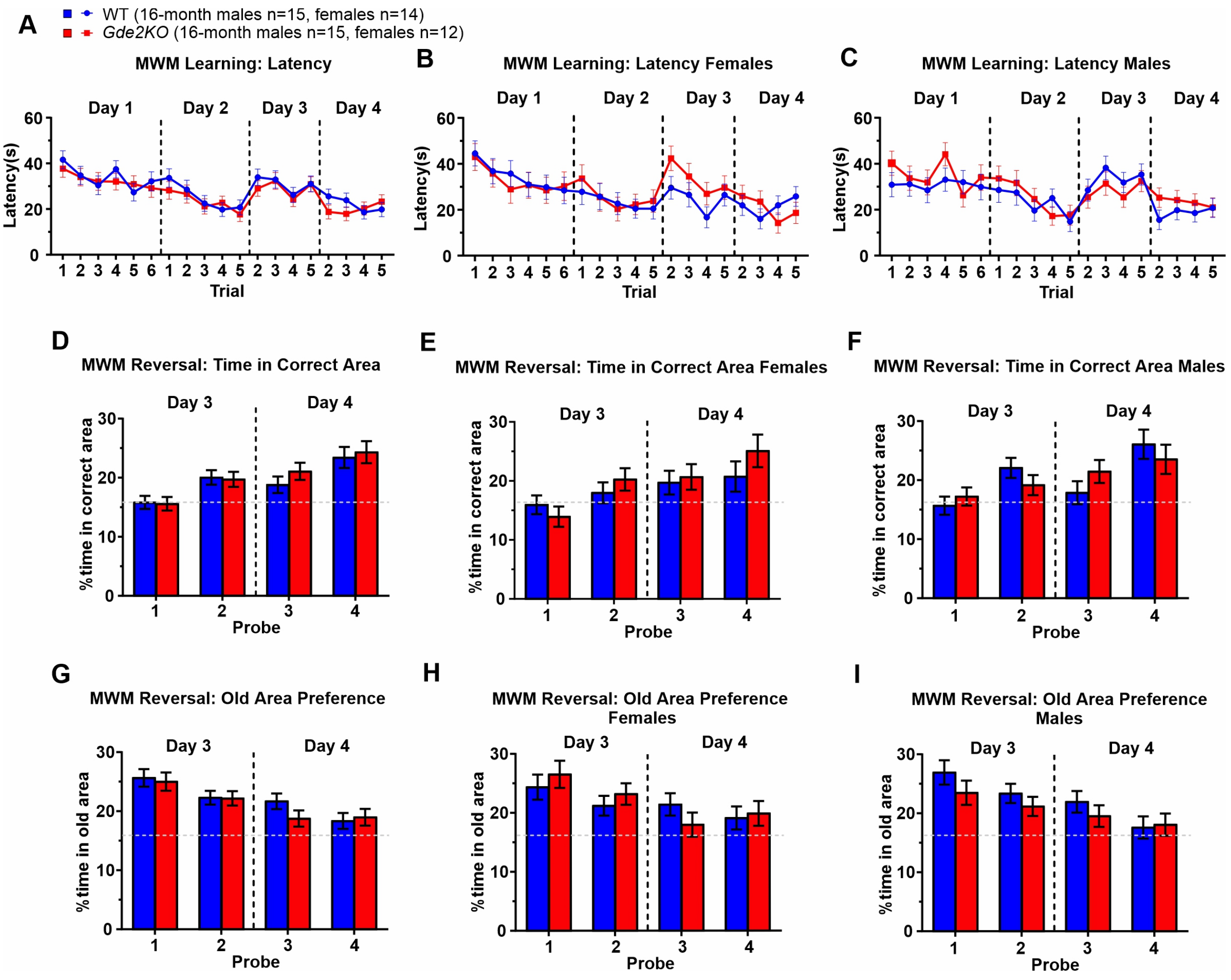

Fig. S8: *Gde2*KO mice learn new platform location during reversal days

(A-C) Latency of training trials during the 4-day MWM task for WT and *Gde2*KO mice with sex combined (A), and separated by females (B) and males (C). (D-F) Analysis of the percent time spent by mice in quadrant 3 platform area during day 3 and 4 probe trials where sex has been combined (D) and separated into females (E) and males (F). (G-I) Quantification of the percent time spent by mice in quadrant 1 platform area during day 3 and 4 probe trials where sex has been combined (G) and separated into females (H) and males (I). No effect of genotype or its interactions was detected for all tests in this figure (three-way mixed design ANOVA, Ps>0.05). All graphs are means ± SEM; ns, $P > 0.05$; $^{*}P < 0.05$, $^{**}P < 0.01$, $^{***}P < 0.001$, and $^{****}P < 0.0001$. See Table S1 for statistical details.

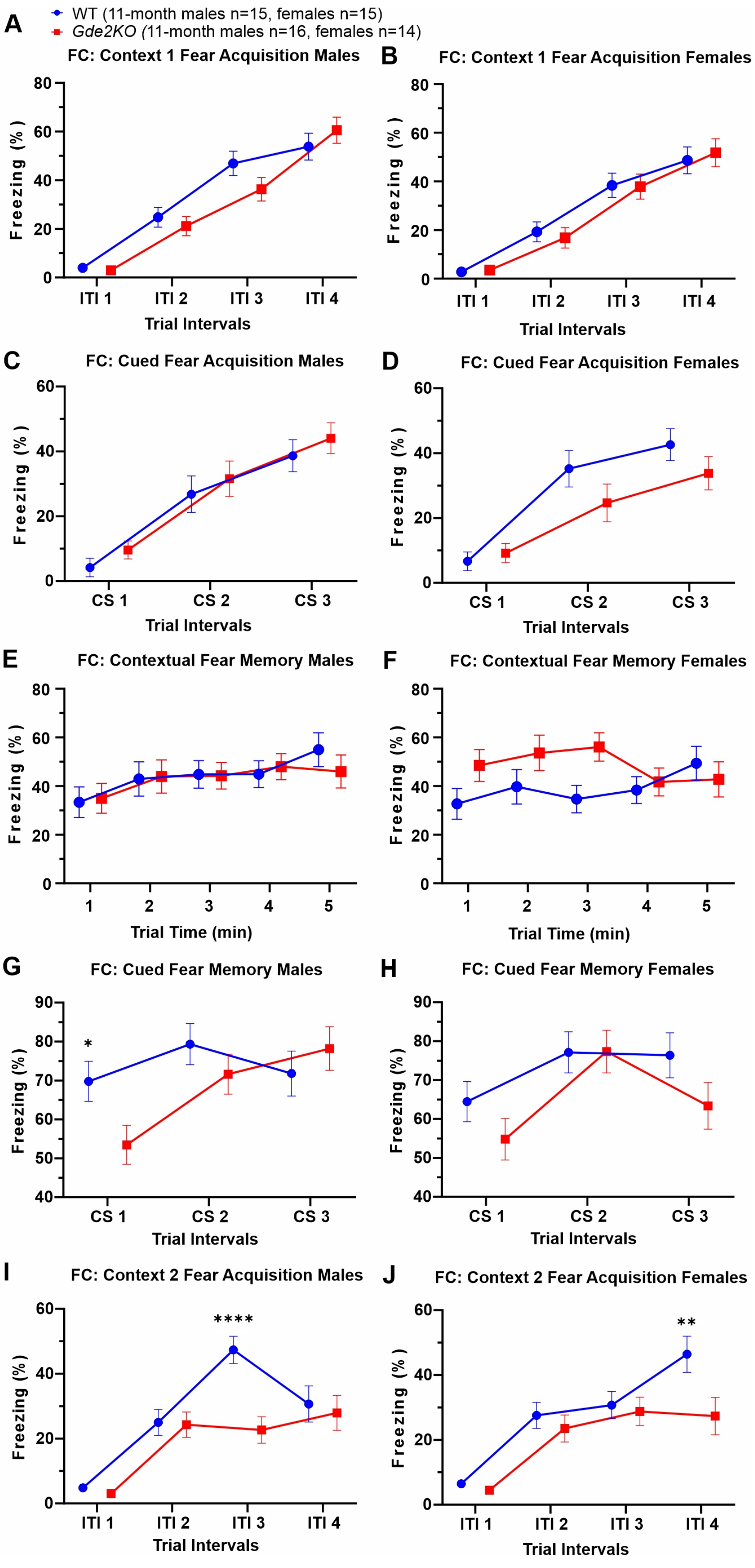

WT (11-month males n=15, females n=15)
Gde2KO (11-month males n=16, females n=14)
A
FC: Context 1 Fear Acquisition Males
Freezing (%)
ITI 1
ITI 2
ITI 3
ITI 4
Trial Intervals
B
FC: Context 1 Fear Acquisition Females
C
FC: Cued Fear Acquisition Males
CS 1
CS 2
CS 3
D
FC: Cued Fear Acquisition Females
E
FC: Contextual Fear Memory Males
Trial Time (min)
F
FC: Contextual Fear Memory Females
G
FC: Cued Fear Memory Males
*
H
FC: Cued Fear Memory Females
I
FC: Context 2 Fear Acquisition Males
****
J
FC: Context 2 Fear Acquisition Females
**

Fig. S9: Sex-separated analysis of FC task

(A-D) Analysis of context 1 fear acquisition and cued fear acquisition on trial 1 day 1 during FC task. Percent time WT and *Gde2*KO mice spent freezing in the intertrial intervals (ITI) separated by males (A) and females (B). Analysis of percent time spent freezing during the CS delivery separated by males (C) and females (D). No effect of genotype or its interactions was detected for tests in trial 1 (three-way mixed design ANOVA, Ps>0.05). (E-F) Dynamics of percent time spent freezing during trial 2 day 2 of FC task separated by males (E) and females (F). No effect of genotype or its interactions was detected for tests in trial 2 (ANOVA, Ps>0.05). (G-J) Analysis of context 2 fear acquisition and cued fear memory during trial 3 day 2. Percent time spent freezing during the delivery of the CS in trial 3 for males (G) and females (H). Male *Gde2*KO mice froze less than WT mice at the start of trial 3 (Fisher LSD post hoc, P<0.04). Percent time spent freezing for males (I) and females (J) during ITIs in trial 3. Male *Gde2*KO mice froze less than WT mice during ITI 3 (Fisher LSD post hoc, P<0.00003), and female *Gde2*KO mice froze less than WT mice during ITI 4 (P<0.002 Fisher LSD post hoc) All graphs are means ± SEM; ns, $P > 0.05$; *$P < 0.05$, **$P < 0.01$, and ****$P < 0.0001$. See Table S1 for statistical details.
